# Multicomponent Reactions for the Synthesis of Active Pharmaceutical Ingredients

**DOI:** 10.3390/ph15081009

**Published:** 2022-08-17

**Authors:** Ángel Cores, José Clerigué, Emmanuel Orocio-Rodríguez, J. Carlos Menéndez

**Affiliations:** 1 Unidad de Química Orgánica y Farmacéutica, Departamento de Química en Ciencias Farmacéuticas, Facultad de Farmacia, Universidad Complutense, 28040 Madrid, Spain; 2 Complutense University of Madrid, 28040 Madrid, Spain

**Keywords:** Strecker reaction, Mannich reaction, Petasis reaction, Povarov reaction, Hantzsch dihydropyridine synthesis, Hantzsch pyrrole synthesis, Asinger reaction, Passerini reaction, Ugi reaction, Gewald reaction

## Abstract

Multicomponent reactions 9i.e., those that engage three or more starting materials to form a product that contains significant fragments of all of them), have been widely employed in the construction of compound libraries, especially in the context of diversity-oriented synthesis. While relatively less exploited, their use in target-oriented synthesis offers significant advantages in terms of synthetic efficiency. This review provides a critical summary of the use of multicomponent reactions for the preparation of active pharmaceutical principles.

## 1. Introduction

Due to the increasing structural complexity of drug molecules, the production of active pharmaceutical ingredients (APIs) generally involves very high costs due to the large number of synthetic steps often involved and also due to the very stringent purity requirements imposed by regulatory agencies. For the same reasons, API synthesis is also associated with the generation of large amounts of chemical waste, and the E-factor (a green metric defined as the mass of product to mass of waste ratio) associated with the pharmaceutical industry is higher than that corresponding to all other fine chemical industries [1]. To change this situation significantly, API synthesis needs to be made more efficient, particularly in terms of step economy and avoidance of intermediate isolation and purification steps.

Most current strategies for drug synthesis are based on linear schemes, which are rather inefficient in terms of time, costs, and waste generation. One way to improve this situation would be the introduction of multiple bond-forming transformations as key steps in drug synthesis, since they are usually able to generate high molecular complexity in a single step, representing significant progress towards the goal of the “ideal synthesis” [2]. Among them, multicomponent reactions (MCRs), i.e., those that involve at least three starting materials that contribute all or most of their atoms to the final product, have attracted much attention in recent years. Their advantages over multistep protocols include a higher synthetic efficiency and a reduction in waste generation due to their high atom- and step-economy, which allows for a lower consumption of solvents in the extraction and purification stages.

In this context, we summarise here the progress made towards the preparation of active pharmaceutical ingredients by routes that involve a multicomponent reaction as the key step. Although there have been some previous reviews on the use of MCRs in the synthesis of bioactive compounds in general [3,4,5,6,7,8], to our knowledge this is the first one to comprehensively focus on API preparation. We have organised the material according to the reaction that constitutes the key step of each synthesis and, with very few exceptions, we discuss only compounds that have at least reached clinical investigation status.

## 2. Imine-Initiated Multicomponent Reactions

### 2.1. Strecker Reaction

The reaction between amines, aldehydes, or ketones and a cyanide salt is one of the classical reactions in organic chemistry and holds the distinction of being the first multicomponent reaction described in the literature. It was reported in 1850 by Adolph Strecker, who described the reaction of an aqueous solution of ammonia, acetaldehyde and hydrogen cyanide to yield an α-amino nitrile derivative, whose hydrolysis afforded alanine. Remarkably, this was also the first synthesis of an amino acid, achieved before the availability of methods to isolate them from natural sources. The Strecker reaction is still a valuable tool to prepare natural and non-natural amino acids, as well as intermediates in the synthesis of complex heterocycles, including natural products [9]. Some methodologies that have been shown to afford improvements of the Strecker reaction include ultrasound irradiation [10] and mechanochemical activation [11].

**Scheme 1:**
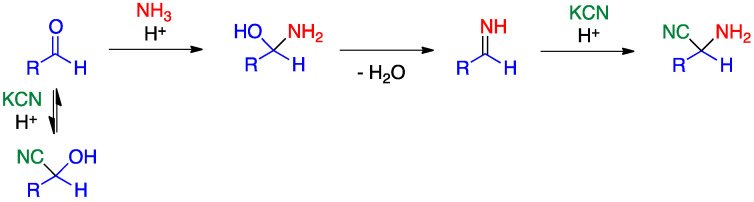
The Strecker reaction and its mechanism.

The commonly accepted mechanism of the Strecker reaction (Scheme 1) starts with the acid-promoted condensation of the amine with the carbonyl compound, affording an imine species (protonated in the acidic medium) with concomitant loss of a water molecule. The cyanide group then attacks the iminium intermediate through a Mannich-like mechanism to give the corresponding α-amino nitrile. In the initial step, a competition exists between cyanide and ammonia for the electrophilic carbonyl carbon, and the kinetic product is a cyanohydrin. However, its formation is reversible under the reaction conditions and the equilibria are eventually displaced towards α-aminonitrile formation [12]. 

**Scheme 2:**
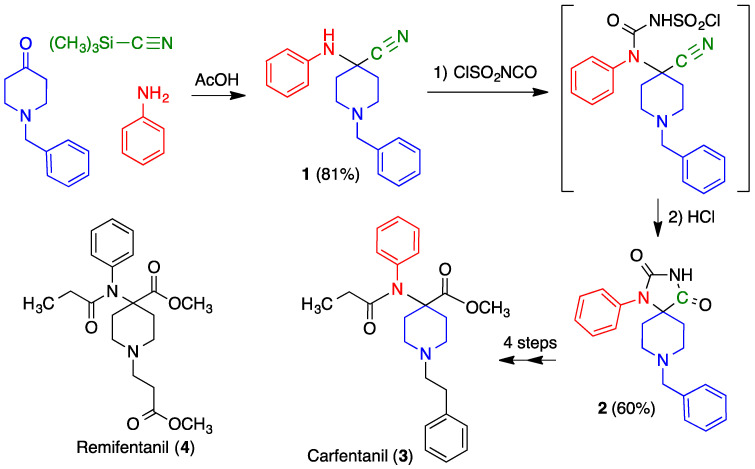
Synthesis of carfentanil based on a Strecker reaction.

The Strecker reaction represents a useful tool for the synthesis of some families of opioid analgesics that are structurally characterised by the presence of a 4-aminopiperidine-4-carboxylic ester moiety. For example, early in the 1990s, Feldman and Brackeen published a simple synthesis of carfentanil (**3**), an opioid 27 times more potent than fentanyl used in veterinary medicine and as a recreational drug, based on this chemistry [13]. The route was initiated by the reaction of *N*-benzyl-4-piperidinone with aniline and trimethylsilyl cyanide, which furnished the racemic α-aminonitrile **1**. This reaction was found to proceed in better yields when performed in anhydrous conditions and in the presence of glacial acetic acid due to the low stability of **1** in aqueous acidic conditions. A six-step route, involving the formation of an hydantoin intermediate **2,** completed the synthesis of carfentanil. Remifentanil **4**, a related opiate analgesic, has been obtained using the same strategy (Scheme 2).

**Scheme 3:**
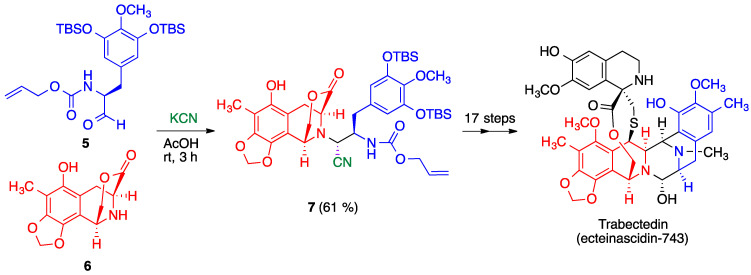
Total synthesis of trabectedin by Corey, having a Strecker reaction as one of the key steps.

Most α-aminonitriles of relevance as intermediates in API synthesis are chiral, and their preparation in enantiomerically pure form is often achieved by diastereoselective Strecker reactions starting from chiral starting materials. One example can be found in the first total synthesis, unveiled by the Corey group, of the anticancer natural product trabectedin (ecteinascidin-743), commercialised under the brand name Yondelis^®^ for the treatment of advanced soft-tissue sarcoma and ovarian cancer. The chemical structure of this alkaloid is highly complex, since in addition to the characteristic pentacyclic template of the tetrahydroisoquinoline family of natural products, a further tetrahydroisoquinoline moiety is attached to the B-ring by a 10-linked lactone. As shown in Scheme 3, the reaction of aldehyde **5** and tetrahydroisoquinoline derivative **6** in the presence of potassium cyanide using acetic acid afforded α-aminonitrile **7**, from which the complex polycyclic scaffold of trabectedin was built in 17 additional steps [14].

**Scheme 4:**
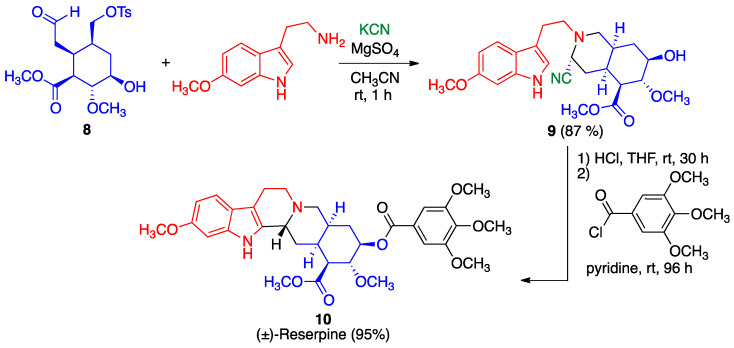
Total synthesis of (±)-reserpine by Stork.

An early example of a bioactive natural product synthesis based on a Strecker reaction can be found in the work by Stork et al. on reserpine, a natural antiadrenergic compound introduced in clinical practice in the 1950s as an antihypertensive agent. Although, due to safety issues, it has been progressively replaced by newer drugs, it is still employed, in combination with diuretics, when more usual treatments fail. A diastereoselective synthesis of this drug, in racemic form, has as the key step the treatment of aldehyde tosylate **8** with 6-methoxytryptamine and potassium cyanide, which led to the formation of cyanopiperidine **9** in high yield. This Strecker product was transformed into (±)-reserpine **10** by a simple Pictet-Spengler cyclisation in acidic conditions, with concomitant cyanide elimination [15] (Scheme 4). 

**Scheme 5:**
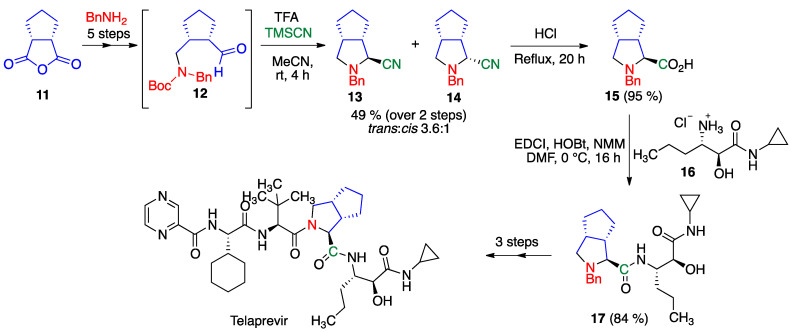
Synthesis of telaprevir based on an intramolecular Strecker reaction.

Although it is not strictly a multicomponent reaction since two partners (the amine and carbonyl groups) are part of the same molecule, we will mention one example of an intramolecular Strecker reaction. Sun, Xu, et al. developed a method to synthesise 3,4-disubstituted proline derivatives serving as suitable substrates for intramolecular Strecker reactions and applied this strategy to the synthesis of the hepatitis C virus protease inhibitor telaprevir [16]. Thus, a five-step route starting from anhydride **11** afforded the *N*-protected aldehyde intermediate **12**, which was reacted with trifluoroacetic acid to deprotect the amine and trimethylsilyl cyanide to furnish the α-aminonitrile product as a mixture of diastereomers **13** and **14**. The hydrolysis of this mixture afforded the corresponding α-amino acid **15** as a single diastereomer, and this compound was coupled with amine **16** to yield **17**, that was converted into telaprevir through a three-step sequence (Scheme 5). This procedure allowed for the synthesis of telaprevir with an overall 16% yield over 11 steps.

**Scheme 6:**
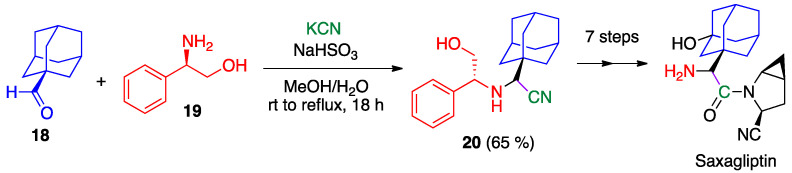
Synthesis of saxagliptin using a chiral auxiliary-based Strecker reaction.

The use of a chiral auxiliary to obtain enantiomerically pure Strecker products is illustrated by the synthesis of saxagliptin, an oral antidiabetic drug belonging to the dipeptidyl peptidase 4 (DPP-IV) inhibitors class. The synthetic route developed by Hamann’s group [17] involves an asymmetric Strecker reaction between the adamantane-derived aldehyde **18**, (*R*)-(−)-2-phenylglycinol **19** and potassium cyanide in the presence of sodium bisulfite, that affords the corresponding homochiral aminonitrile derivative **20** with good yields and excellent enantioselectivity. Seven additional steps, including the removal of the chiral auxiliary, were required to complete the synthesis of saxagliptin (Scheme 6).

**Scheme 7:**
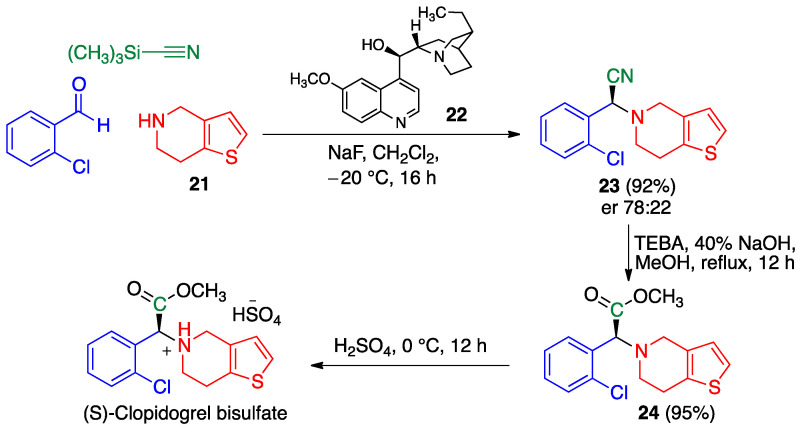
Synthesis of (*S*)-clopidogrel bisulfate based on an organocatalytic, enantioselective Strecker reaction.

Finally, we will discuss the use of chiral catalysts for enantioselective Strecker reactions as key steps in drug synthesis. Bajaj et al. published in 2012 an efficient synthesis of (*S*)-clopidogrel, an antiplatelet drug widely used in the prevention of ischemic events in patients with atherosclerosis, based on a new asymmetric Strecker reaction [18]. Briefly, they optimised a procedure by which different aliphatic or aromatic aldehydes could react with secondary amines and trimethylsilyl cyanide in the presence of a hydroquinine derivative as a chiral catalyst and sodium fluoride to improve the reactivity of (CH_3_)_3_SiCN, yielding the corresponding α-amino nitriles with good yields and enantiomeric excesses. To exemplify the utility of this new protocol, they efficiently transformed 2-chlorobenzaldehyde and 4,5,6,7-tetrahydrothieno[3,2-*c*]pyridine **21,** in the presence of catalyst **22**, into intermediate **23**. This compound was hydrolyzed to the corresponding carboxylic acid **24** and then transformed into (*S*)-clopidogrel bisulfate by a Fischer esterification followed by salt formation (Scheme 7). This synthetic pathway achieved a remarkable shortening of the overall process when compared to the previously available multistep routes [19,20,21].

**Scheme 8:**
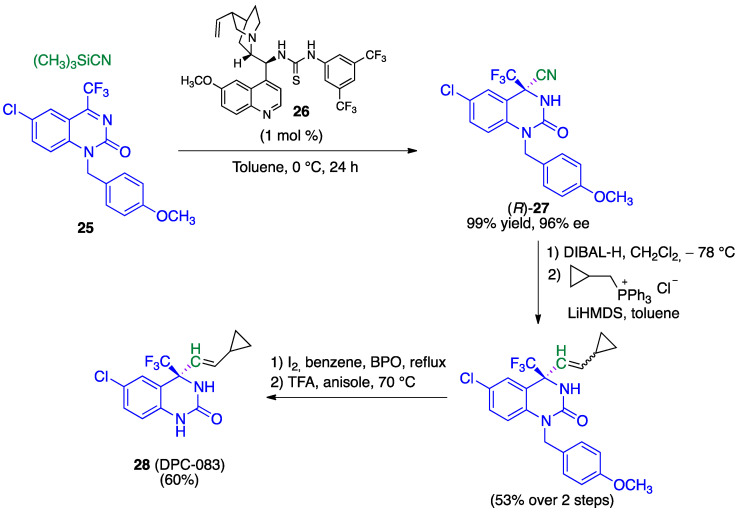
Enantioselective synthesis of the anti-HIV clinical candidate DPC-083.

DPC-083 is a non-nucleoside reverse transcriptase inhibitor that reached phase II clinical trials for the treatment of HIV infection. The group of Ma accomplished its enantioselective synthesis by using an in-house developed organocatalytic Strecker reaction [22]. This protocol starts from imine **25** and trimethylsilyl cyanide in the presence of a bifunctional cinchonine-derived thiourea chiral catalyst (**26**), affording the intermediate (*R*)-**27** with excellent yield and enantioselectivity. Then, reduction of the nitrile group to an aldehyde, a Wittig reaction that gave a *cis* alkene as the major product, double bond isomerization by treatment with iodine followed by benzoyl peroxide (BPO) and a final N-deprotection step allowed the synthesis of DPC-083 **28** in an acceptable overall yield (Scheme 8). No racemization of the quaternary stereogenic center was observed during the last steps of the process.

### 2.2. Bucherer-Bergs Reaction

The reaction of aldehydes or ketones, potassium cyanide and ammonium carbonate to yield 5-substituted or 5,5-disubstituted hydantoins is known as the Bucherer-Bergs reaction [23], after Hermann Bergs, the first chemist who formally described it in a patent [24] and Hans Theodor Bucherer, who developed the conditions and applications of this method [25]. Previously, Ciamician and Silber had observed in 1905 the formation of 5,5-dimethylhydantoin from a mixture of acetone and hydrocyanic acid exposed to sunlight for a period of five to seven months [26]. Although the reaction works well with aliphatic or aromatic aldehydes and most ketones, when compared with other methods for hydantoin synthesis [27], it presents the drawback of showing only one point of variability, the C-5 position. Some modifications have been proposed to overcome reaction failures or improve yields, including ultrasonication of the reaction medium [28], the Hoyer modification, consisting of carrying out the reaction under a CO_2_ atmosphere at high pressures [29], flow chemistry [30] and mechanochemical activation [31].

**Scheme 9:**
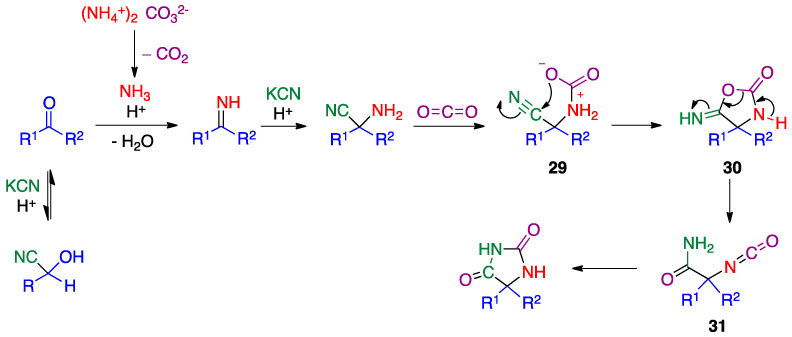
The Bucherer–Bergs reaction and its mechanism.

The Bucherer-Bergs and Strecker reactions start similarly: the carbonyl compound condensates with ammonia, generating an imine suitable to receive an addition of cyanide. The resulting α-amino nitrile reacts with CO_2_, arising from ammonium carbonate, to form a carbamic acid intermediate **29**, which undergoes a spontaneous cyclisation to yield the 5-iminooxazolidin-2-one **30**. Ring opening of this new species gives the corresponding isocyanate **31**, which is transformed into the final hydantoin product through a second cyclisation by addition of the amino group to the isocyanate [23] (Scheme 9).

**Scheme 10:**
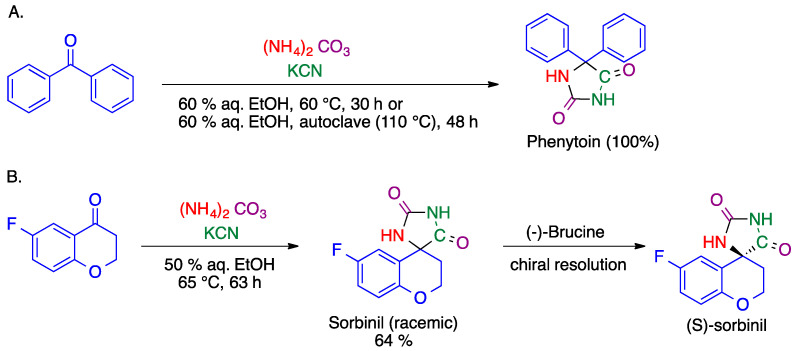
Two active pharmaceutical ingredients prepared by the Bucherer–Bergs reaction: (**A**). The antiepileptic drug phenytoin. (**B**). The aldose reductase inhibitor sorbinil.

The most recognizable API that can be obtained through a Bucherer–Bergs reaction is phenytoin. The multicomponent synthesis of this popular drug, widely used as an antiepileptic drug, was first described in a patent from 1946 by reaction between benzophenone, ammonium carbonate and potassium cyanide in 60% aqueous ethanol. Alternatively, the reaction of benzophenone, potassium cyanide, and ammonium carbonate in an autoclave at 110 °C for 48 h gave phenytoin in nearly quantitative yield (Scheme 10A). According to the patent, other solvents at least partially miscible with water can be used, including aliphatic amides, alkyl glycols, dioxane, etc., and other cyanide salts, such as sodium, lithium or calcium cyanide, are also compatible with the method [32].

Sorbinil is an aldose reductase inhibitor that has been evaluated in clinical trials to treat complications of chronic diabetes such as diabetic neuropathy. An U.S. patent of 1984 describes a simple method for its synthesis, where the key step involves a Bucherer–Bergs reaction from 6-fluoro-4-chromanone, potassium cyanide and an excess of ammonium carbonate in aqueous ethanol at 65 °C for 63 h. An additional step of racemic resolution allowed the isolation of the pharmacologically active (*S*)-(+)-enantiomer [33] (Scheme 10B).

**Scheme 11:**
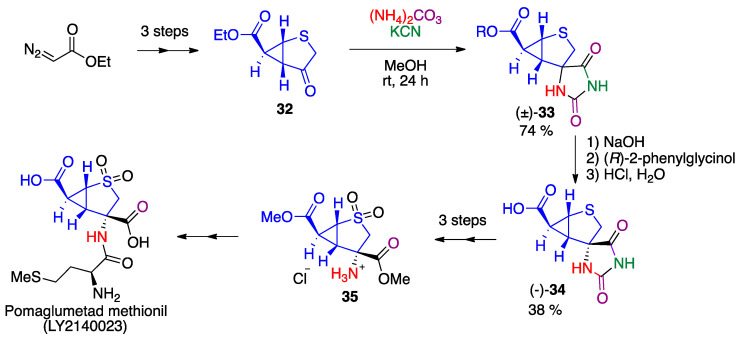
Synthesis of pomaglumetad methionil.

Pomaglumetad methionil (also called LY2140023) is a methionine amide of pomaglumetad, a non-natural amino acid that exhibits a potent agonist activity on mGlu2/3 receptors, particularly abundant in limbic and forebrain areas. It was proposed for the treatment of schizophrenia but failed to show efficacy in phase III clinical trials, although it may be effective in certain patient populations [34]. Waer, Moher, et al. proposed a highly scalable synthetic pathway for this drug (Scheme 11) that starts from ethyl diazoacetate, which was transformed into ketone **32** by a three-step route. Compound **32** was reacted with ammonium carbonate and potassium cyanide under the classical Bucherer–Bergs conditions, yielding a mixture of methyl and ethyl esters, due to transesterification reactions, that was saponified to afford the desired hydantoin **33**. During optimization, it was assessed that in the Bucherer–Bergs reaction 1 equivalent of KCN was enough to achieve the optimal yield of the product and 3.5 equivalents of ammonia guaranteed the robustness of the procedure. Compound **34**, a key intermediate for the synthesis of LY2140023, was isolated after the enantiomeric resolution of **33**. Hydrolysis of the hydantoin framework followed by two additional steps provided the amino acid **35**, which was finally transformed into pomaglumetad methionil (LY2140023) [35].

### 2.3. Mannich Reaction

**Scheme 12:**
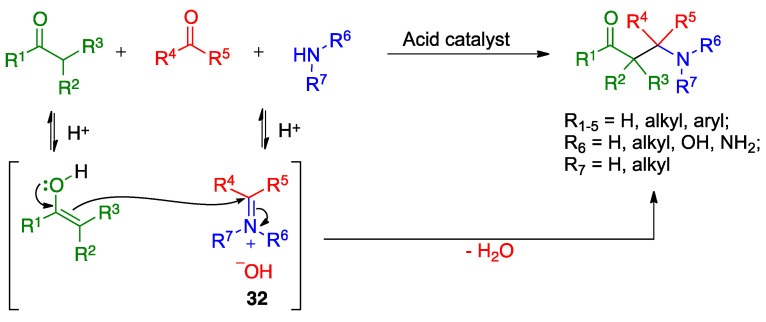
The acid-catalyzed Mannich reaction and its mechanism.

The Mannich reaction starts from ammonia or a primary or secondary amine, generally as a hydrochloride salt, an aldehyde and a compound having at least one active hydrogen atom to provide aminomethyl derivatives of the latter compound [36]. The Mannich reaction can take place either under basic or acidic conditions, although the latter are more common (Scheme 12). Under acidic conditions, the amine first reacts with the non-enolizable carbonyl compound to give a hemiaminal that after dehydration leads to an iminium cation **32**. This species reacts with the enolizable carbonyl compound through an aldol-type reaction resulting in the formation of the final product.

**Figure 1:**
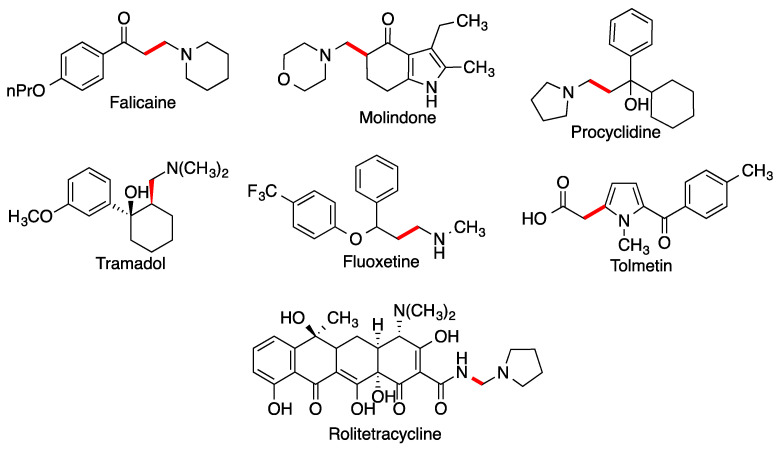
Some drugs that are synthesised using a Mannich reaction as the key step. The bond thus created is marked in red.

While the first example of this transformation was reported in 1903 by Tollens and von Marle, it was not until 1912 that Carl Mannich recognised its generality as a method for the synthesis of alkylated β-ketoamines (known as Mannich bases) and started its systematic study. Nowadays, the Mannich reaction, as well as its asymmetric variations [37], has an important role in organic synthesis, including the preparation of complex natural products [38,39] and bioactive molecules [6]. Many drug molecules contain structural fragments derived from a Mannich reaction or from reaction sequences initiated by a Mannich reaction (e.g., Mannich/Grignard); some representative examples are shown in Figure 1.

**Scheme 13:**
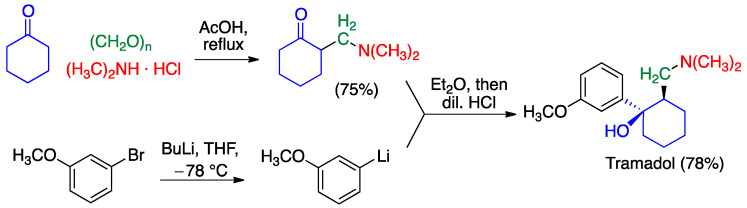
Synthesis of tramadol.

As a representative example, we show in Scheme 13 the synthesis of the analgesic drug tramadol, which involves an initial Mannich reaction between cyclohexanone, formaldehyde and dimethylamine followed by treatment of the resulting Mannich base with 3-methyoxyphenlyllithium [40]. A similar sequence, using a Grignard reagent instead of the phenyllithium derivative, has allowed for a continuous-flow synthesis of tramadol [41].

**Scheme 14:**
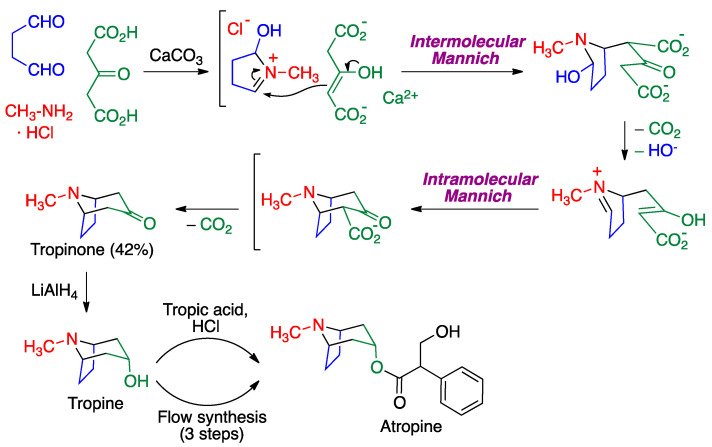
The Robinson tropinone synthesis and its application to the preparation of atropine.

Double Mannich reactions have been successfully employed for the synthesis of six-membered nitrogen heterocyclic rings. Sir Robert Robinson’s pioneering synthesis of tropinone [42] involved a multicomponent domino process comprising inter- and intramolecular Mannich steps (Scheme 14). This reaction is historically relevant in that it constituted the first example of a biomimetic synthesis (i.e., a synthetic route designed to imitate a biosynthetic pathway). Reduction of tropinone provides tropine, a key building block in the synthesis of bioactive tropane alkaloids. For instance, atropine, an antimuscarinic alkaloid present in the “Essential Drugs List” by the World Health Organization, is employed to treat bradycardia and poisoning by organophosphate pesticides acting as acetylcholinesterase inhibitors. This compound can be prepared by Fischer esterification of tropine with tropic acid or with methyl formylphenylacetate, followed by NaBH_4_ reduction of the formyl group [43]. A related three-step flow protocol for atropine synthesis has been developed [44].

**Scheme 15:**
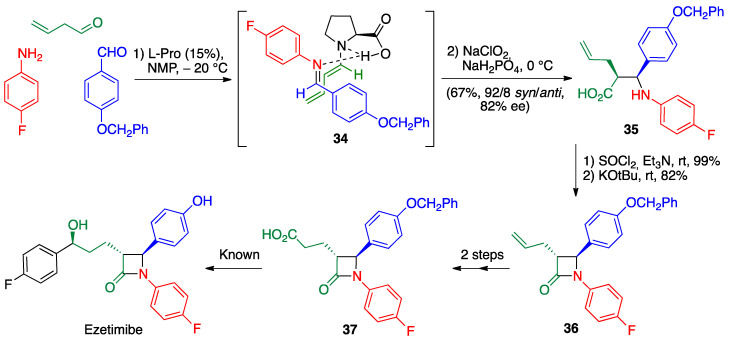
Enantioselective synthesis of ezetimibe based on an organocatalytic Mannich reaction.

Asymmetric Mannich reactions are also important in drug synthesis. Ezetimibe is an inhibitor of the NPC1L1 transporter, responsible for the intestinal absorption of cholesterol and phytosterols, and it is an approved treatment of hypercholesterolemia for the prevention of cardiovascular events. The β-lactam moiety in this compound makes it particularly amenable to be prepared via the Staudinger [2 + 2] ketene-imine cycloaddition [45], but this reaction is not normally carried out in a multicomponent fashion [46] and will not be discussed here. On the other hand, Hayashi has described [47] an enantioselective formal total synthesis of this drug based on the proline-catalyzed Mannich reaction of 3-butenal, 4-fluoroaniline and 4-benzyloxybenzaldehyde by way of transition state **34**. The resulting Mannich base was oxidised to **35**, and then cyclised to the corresponding β-lactam and submitted to inversion of its stereocenter α to carbonyl by treatment with base, yielding compound **36**. Two additional steps allowed the transformation of the latter compound into **37**, a previously known precursor to ezetimibe (Scheme 15).

**Scheme 16:**
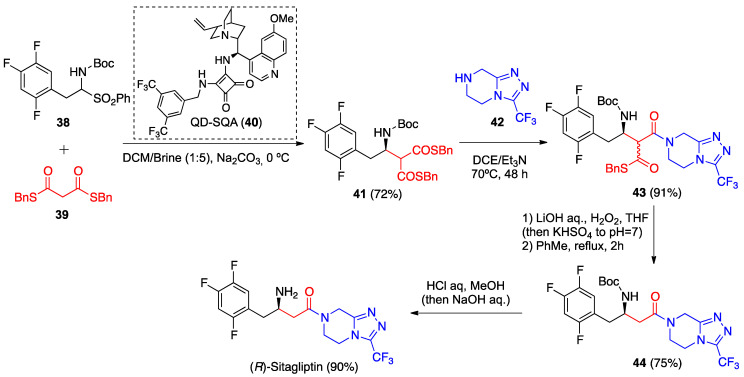
An asymmetric, organocatalytic version of the Mannich reaction and its application to the synthesis of the antidiabetic drug (*R*)-sitagliptin.

Asymmetric Mannich reactions are often performed from isolated imines. In 2016, Song and co-workers developed an enantioselective Mannich reaction able to work on alkylimines in the presence of a quinine-derived squaramide (QD-SQA) chiral catalyst and applied it to the synthesis of (*R*)-(−)-sitagliptin, a commercially available antidiabetic drug [48]. Thus, a non-isolated imine generated in situ from *N*-protected α-amidosulphone **38** reacted with dithiomalonate **39** in the presence of QD-SQA (**40**), giving compound **41** in good yield (72%) and excellent enantiomeric excess (>99%). Then, **41** was coupled with the [1,2,4]triazolo[4,3-*a*]pyrazine derivative **42**, corresponding to the secondary amine component, to provide **43** without the need for a coupling reagent. Hydrolysis of the thioester group and decarboxylation of the resulting carboxylic acid allowed the formation of a N-Boc derivative of sitagliptin (**44**). Finally, the Boc group was removed under acidic conditions to yield the desired drug (Scheme 16).

### 2.4. Petasis (Borono-Mannich) Reaction

**Scheme 17:**
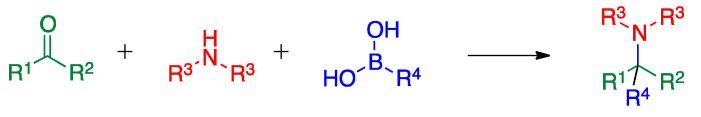
The Petasis (borono-Mannich) reaction.

In 1993, Nicos Petasis and Irini Akritopoulou reported a variation of the Mannich reaction that affords allylic amines from aldehydes, amines, and boronic acids, which act as the nucleophilic component (Scheme 17). It is a selective and mild method, compatible with the presence hydroxyl and carboxy groups in the molecule core and able to retain the double bond geometry when vinylboronic acids are employed [49]. Mechanistically, the Petasis reaction involves the formation of an iminium ion by condensation between the amine and the carbonyl compound. This intermediate then coordinates with the boronic acid to facilitate the migration of the substituent attached to the boron atom to the iminium carbon [50].

**Scheme 18:**
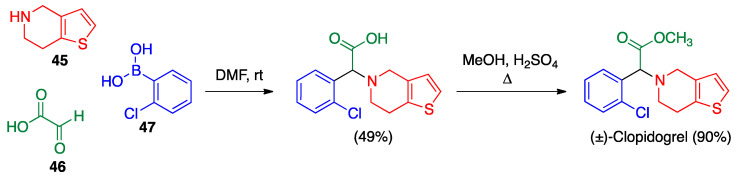
Synthesis of racemic clopidogrel based on a Petasis reaction.

As an example of the use of the Petasis reaction in drug synthesis, racemic clopidogrel has been prepared in overall good yield, using a three-component Petasis reaction from tetrahydrothieno[3,2-*c*]pyridine derivative **45**, glyoxylic acid **46** and boronic acid **47**, followed by a Fischer esterification with methanol (Scheme 18) [51].

**Scheme 19:**
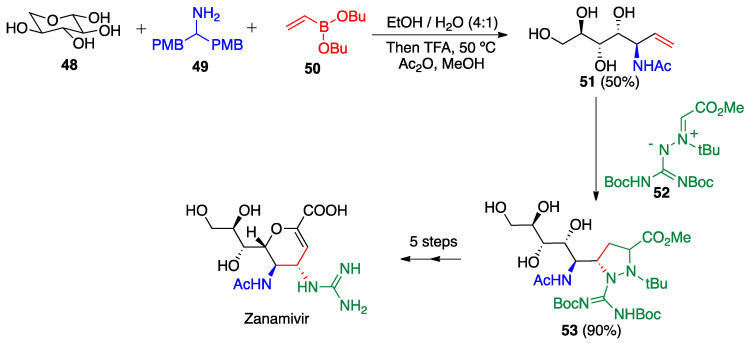
Synthesis of zanamivir based on a Petasis reaction.

Zanamivir is a potent and highly selective inhibitor of the influenza A and B virus neuraminidase. As shown in Scheme 19, it can be prepared using as the first step a Petasis reaction from D-arabinose (**48**), a protected primary amine (**49**) and a vinylboronic ester (**50**), resulting in the polyhydroxy compound **51**. The next step is a 1,3-dipolar cycloaddition reaction of the terminal olefin in **51** with the 1,3-dipole **52**, and furnishes compound **53**, from which the antiviral drug is obtained after six additional steps [52].

### 2.5. Povarov Reaction

The Povarov reaction is a versatile and efficient method to access the tetrahydroquinoline scaffold. The first examples of this reaction were published by the Russian chemist L. S. Povarov in the 1960s, although it was in the 1990s when its full potential began to be exploited. This process is usually described as an inverse electron-demand hetero Diels-Alder cycloaddition between *N*-arylimines and electron-rich olefins catalyzed by Lewis and Brönsted acids, ionic liquids, resins, etc. A large variety of dienes and dienophiles are suitable substrates for this reaction, and enantioselective variations have been thoroughly explored [53,54]. The original Povarov reaction started from preformed *N*-arylimines and olefins, but the reaction can often be performed as a multicomponent process (i.e., employing an aniline an aldehyde, with the olefin acting as the dienophile). This multicomponent variant has been sometimes referred to as the Grieco reaction.

**Scheme 20:**
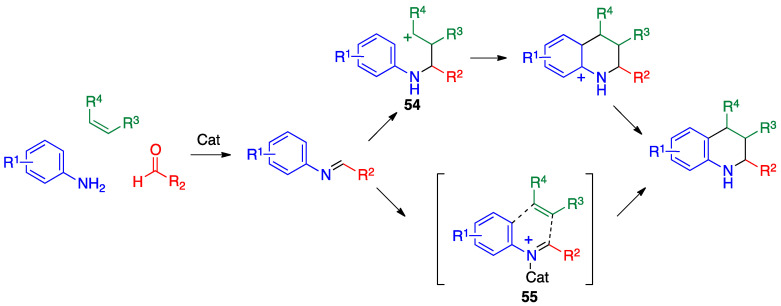
The Povarov reactions and its more relevant mechanistic proposals.

Regarding the Povarov reaction mechanism, chemists working on this field are divided around two hypotheses (Scheme 20). One of them involves a stepwise process, in which the dienophile would perform first a Mannich addition to the activated imine, forming an intermediate **54** that would undergo an intramolecular Friedel-Crafts alkylation and, finally, a re-aromatization step by a [1,3]-hydrogen shift to afford the tetrahydroquinoline derivative [55,56,57]. Some published work would support the formation of **55** by showing its trapping by addition of a nucleophile to the reaction medium [58,59]. The second hypothesis proposes that the Mannich/Friedel Crafts process take place in an asynchronous concerted manner through an ephemeral transition state **55** [60].

**Scheme 21:**
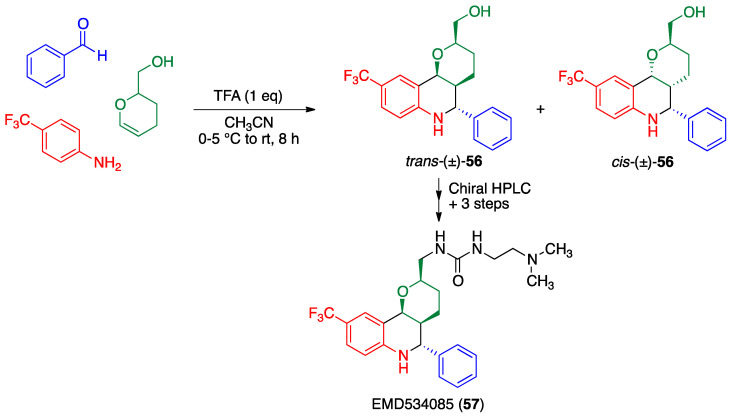
Synthesis of EMD534085.

The Povarov tetrahydroquinoline synthesis has been useful to synthesise drug candidates, EMD534085 being a relevant example. This molecule is a potent inhibitor of Eg5, a kinesin related motor protein, causing mitotic arrest. It is widely employed as a tool for biochemical research, and it has been evaluated in a phase I clinical trial for the treatment of solid tumors and lymphoma. Its synthesis, reported by Schiemann et al. [61], starts from the reaction of *p*-trifluoromethylaniline, benzaldehyde and 3,4-dihydro-2*H*-pyran-2-methanol as the dienophile, catalyzed by trifluoroacetic acid. After chiral HPLC for isolating the required enantiomer, the hydroxy group of tetrahydroquinoline **56** was activated and substituted to afford the target EMD534085, maintaining enantiomeric purity (Scheme 21).

**Scheme 22:**
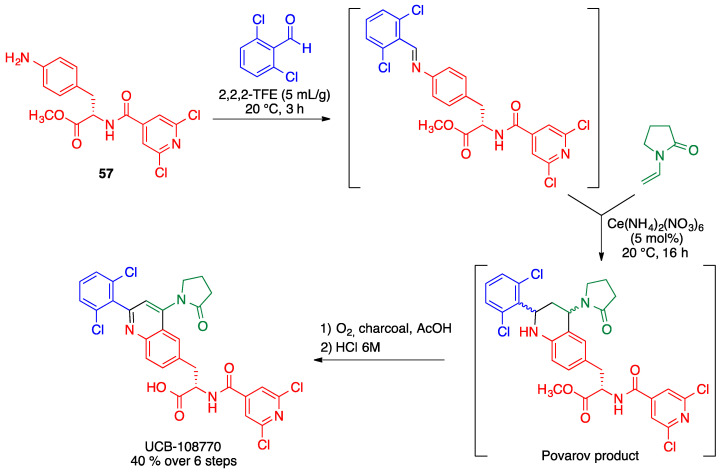
Synthesis of UCB-108770 based on a Povarov reaction.

A Povarov reaction followed by a dehydrogenation step has been employed for the synthesis of UCB-108770, an interesting VLA-4 antagonist potentially useful for the treatment of inflammatory diseases. This compound was synthesised in a 30 kg scale by a route having as the key step a sequential Povarov reaction starting from phenylalanine derivative **57**, 2,6-dichlorobenzaldehyde and *N*-vinyl-2-pyrrolidone in the presence of ammonium cerium (IV) nitrate. The resulting Povarov adduct, in crude form, was directly dehydrogenated to a quinoline derivative by treatment with oxygen in the presence of charcoal. A final hydrolysis of the ester group afforded the final compound UCB-108770 with a 40% global yield over six steps [62] (Scheme 22).

**Scheme 23:**
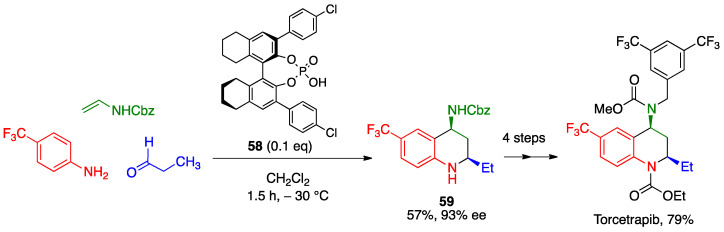
Enantioselective synthesis of torcetrapib.

Torcetrapib is another remarkable example of a drug candidate that can be prepared by a route involving a Povarov reaction as the key step. This compound is an inhibitor of cholesterylester transfer protein, implicated in the metabolism of cholesterol, and it reached phase III clinical trials for the treatment of dyslipidemia although it failed due to safety issues. The group of Zhu and Masson, in 2009, took advantage of their work on the use of *N*-vinyl carbamates as dienophiles for the Povarov reaction to develop a simple procedure for the enantioselective synthesis of torcetrapib [63]. Thus, they employed *p*-trifluoromethylaniline, propionaldehyde and benzyl *N*-vinylcarbamate to obtain the tetrahydroquinoline **59** in good yield and enantioselectivity, using the octahydro-(*R*)-BINOL-derived phosphoric acid **58** as a chiral catalyst. Four additional reaction steps, with an overall yield of 32%, were necessary to achieve the synthesis of torcetrapib (Scheme 23). The same group reported later an improved overall yield of 40% by simply optimizing the reaction time of the Povarov step [64].

### 2.6. Doebner Reaction

**Scheme 24:**
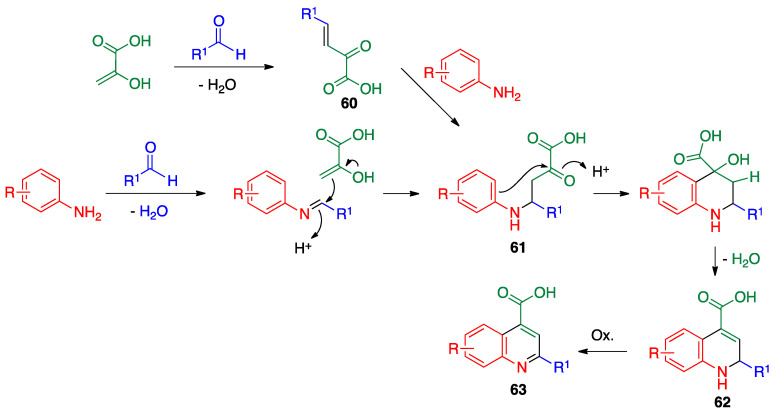
The Doebner quinoline synthesis.

The Doebner reaction is a classical method for the synthesis of quinoline-4-carboxylic acids, starting from an aromatic amine, an aldehyde and pyruvic acid. The reaction was discovered by the German chemist Gustav Doebner in 1884 [65], and it still remains a useful tool to synthesise quinoline derivatives, either by the original methodology or by some modified protocols that have been developed to overcome its drawbacks, such as low yields or harsh reaction conditions. Two main alternatives have been proposed for its mechanism [66]. The first one starts with the aldol condensation of the enol form of pyruvic acid with the aldehyde forming intermediate **60**, with a α,β-unsaturated carbonyl motif that reacts with the aniline via a Michael addition to give **61**, that would undergo dehydrative cyclisation to afford dihydroquinoline **62**, whose air oxidation finally furnishes compound **63**. Alternatively, **61** can be reached by condensation of the aniline with the aldehyde followed by Mannich addition of the enol form of pyruvic acid (Scheme 24).

**Scheme 25:**
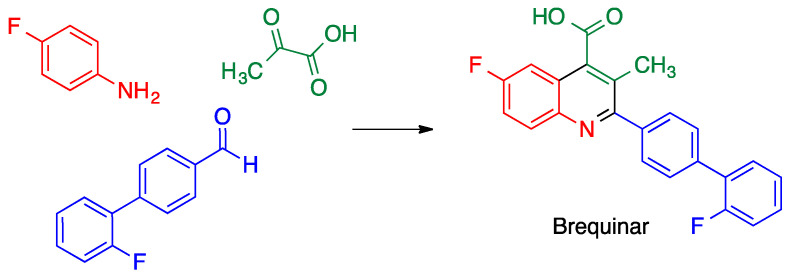
Multicomponent synthesis of brequinar.

Brequinar is an inhibitor of dihydroorotate dehydrogenase (DHODH), a key enzyme in the de novo synthesis of pyrimidine-based nucleotides. Its antiproliferative and immunosuppressant activities have prompted its evaluation in several clinical trials for the treatment of some types of cancer and infections such as malaria, and as an immnunosuppresant for the prevention of rejection after organ transplant. One approach to its synthesis involves a multicomponent Doebner reaction from pyruvic acid, 4-fluoroaniline, and 4-(2′-fluorophenyl)benzaldehyde (Scheme 25) [67].

## 3. Enamine-Initiated Multicomponent Reactions

### 3.1. Hantzsch Dihydropyridine Synthesis

**Scheme 26:**
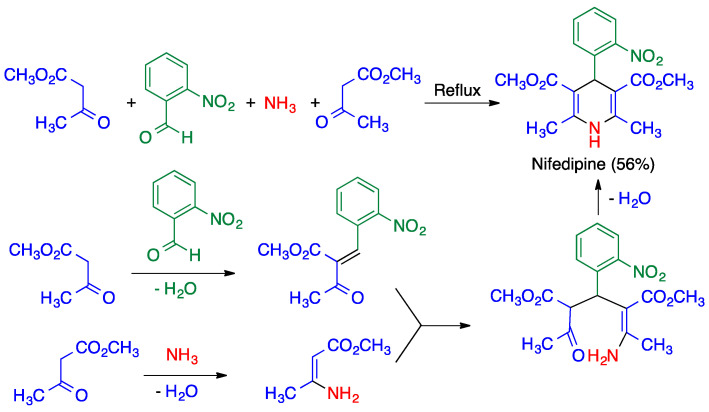
The Hantzsch dihydropyridine synthesis, applied to the preparation of nifedipine.

The Hantzsch dihydropyridine synthesis, discovered by Arthur Rudolf Hantzsch in 1881, is a pseudo four-component reaction between an aldehyde, 2 equivalents of a β-keto ester and ammonia. Its mechanism involves the combination of a Knoevenagel condensation, an enaminoester formation, a Michael addition and a final cyclocondensation, as shown in Scheme 26 for the synthesis of nifedipine [68], a vasodilator used to treat high blood pressure and to control *angina pectoris*.

**Scheme 27:**
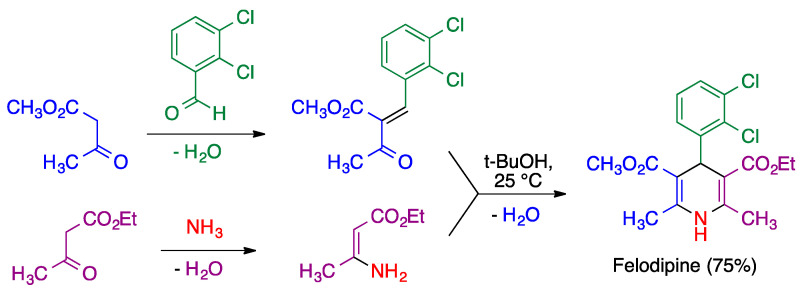
Synthesis of felodipine, a non-symmetrical dihydropyridine.

In order to obtain non-symmetrical dihydropyridines (e.g., felodipine), the two steps of the above mechanism (i.e., the Knoevenagel and enaminoester formation steps), need to be reproduced separately and the resulting intermediates then combined [69] (Scheme 27).

### 3.2. Hantzsch Pyrrole Synthesis

**Scheme 28:**
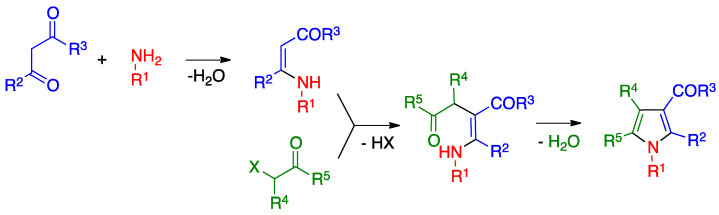
The Hantzsch pyrrole synthesis and its commonly accepted mechanism.

Arthur Rudolf Hantzsch, in 1890, published a note reporting that the reaction of α-chloroacetone, ethyl acetoacetate and aqueous ammonia gave ethyl 2,5-dimethylpyrrole-3-carboxylate. The usually accepted mechanism for this three-component reaction between primary amines, β-dicarbonyl compounds and α-haloketones or aldehydes is shown in Scheme 28 [70].

**Scheme 29:**
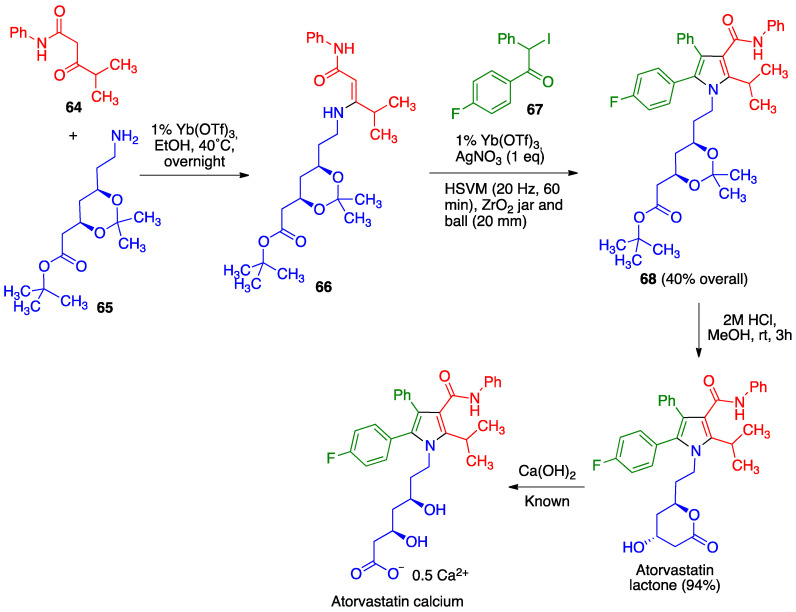
Application of the mechanochemical Hantzsch pyrrole synthesis to the preparation of atorvastatin.

We reported in 2013 that the Hantzsch pyrrole synthesis can be performed under solvent-free conditions, using the high-speed vibration milling (HSVM) technique. Moreover, this solid-state method gave pyrroles in higher yields than previous versions of the Hantzsch reaction and was far broader in scope [71,72]. On this basis, we also reported a short synthesis of atorvastatin, a member of the statins, the main family of cholesterol-lowering drugs. The calcium salt of this compound was marketed in 1997 as Lipitor^®^ and became the best-selling drug in history. Thus, atorvastatin can be arguably regarded as the most important unnatural pyrrole derivative ever made. The application of our mechanochemical Hantzsch pyrrole synthesis to the formal synthesis of atorvastatin is shown in Scheme 29 and starts with the sequential combination of β-ketoamide **64**, the chiral protected primary amine **65** and α-iodoacetophenone derivative **67** to give pyrrole derivative **68**, by way of the intermediate β-eneminoamide **66**. Hydrolytic deprotection of the acetal and *tert*-butyl ester functions afforded atorvastatin lactone, which had been previously transformed in a single step into atorvastatin calcium [73].

**Scheme 30:**
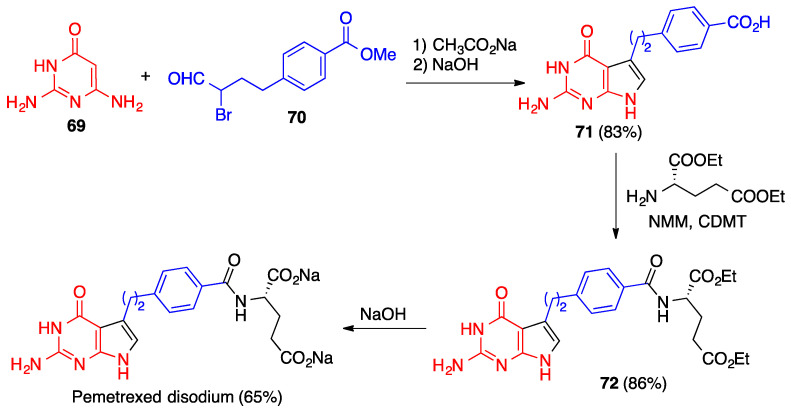
Synthesis of pemetrexed disodium based on a Hantzsch pyrrole synthesis.

A synthesis of the anticancer drug pemetrexed disodium [74] is based on a Hantzsch pyrrole synthesis starting from 2,6-diaminopyrimidin-4(3*H*)-one **69** and α-bromoaldehyde **70**. In the presence of sodium acetate, these starting materials afforded compound **71** after saponification of the ester group. Conjugation with diethyl glutamate to furnish **72** and a final second saponification gave pemetrexed disodium (Scheme 30).

### 3.3. Asinger Reaction

**Scheme 31:**
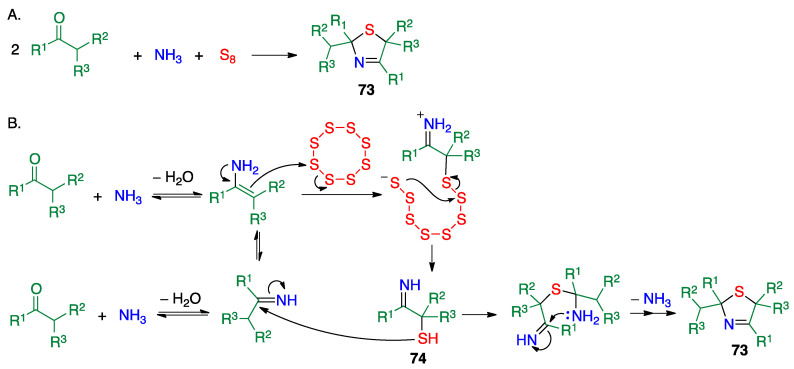
(**A**). The Asinger reaction. (**B**). Its proposed mechanism.

This reaction has two main variations, both discovered by Friedrich Asinger [75]. The first of them is a pseudo-four component reaction from elemental sulfur, gaseous ammonia and two equivalents of a carbonyl compound with at least one acidic α-H atom that yields the thiazoline derivative **73** (Scheme 31A). This process is usually explained by the mechanism summarised in Scheme 31B, involving the formation of both enamine and imine intermediates from the starting carbonyl compound and ammonia. The enamine reacts with sulfur, leading to sulfhydrylimine **74**. This intermediate reacts with the imine and a final ring closure reaction with concomitant ammonia elimination affords thiazolidines **73**.

**Scheme 32:**
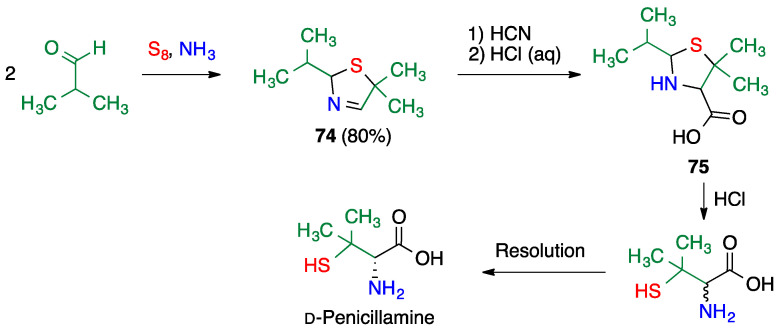
Application of the classical Asinger reaction to the synthesis of D-penicillamine.

As an application of this reaction to API synthesis, we will discuss the synthesis of d-penicillamine, a non-natural amino acid discovered as a degradation product of penicillin. It is an efficient chelating agent and is thus used for the treatment of Wilson’s disease and various heavy metal poisonings, as well as against rheumatoid arthritis [76]. Its synthesis [77] starts with an Asinger reaction from isobutyraldehyde, elemental sulfur and ammonia to form the thiazoline **74**. The Strecker reaction of this compound with HCN afforded a nitrile, which was hydrolyzed to carboxylic acid **75**. The synthesis of d-penicillamine was completed by acid hydrolysis of the thioaminal functional group and racemic resolution (Scheme 32).

**Scheme 33:**
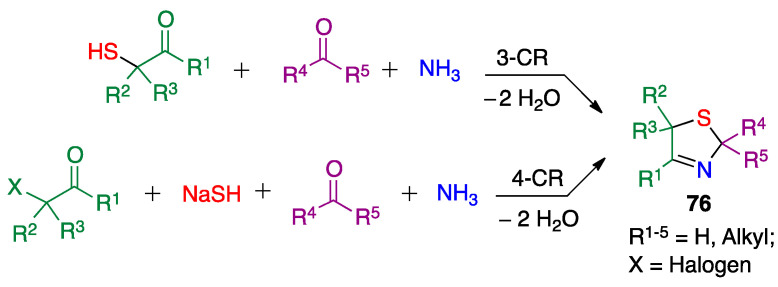
The 3-CR and 4-CR Asinger reactions known as “resynthesis”.

The modified Asinger reaction is known as “resynthesis” and can be defined as a three-component reaction in which an α-mercaptoketone or aldehyde reacts with an aldehyde or ketone and ammonia to form the thiazoline **76**. It can also be a four-component process if the α-mercaptoketone or aldehyde is generated in situ from the corresponding α-halocarbonyl and NaSH (Scheme 33).

An application of this reaction to the synthesis of penicillin derivatives that combines two multicomponent reactions will be discussed in Section 7.

## 4. Isonitrile-Based Multicomponent Reactions

Among the best-known class of multicomponent reactions, those in which an isocyanide (isonitrile) takes part in the reaction are particularly important. These isocyanide-based multicomponent reactions (IMCRs) are among the very first multicomponent reactions to be discovered and are still broadly studied. They have widely broadly employed for API synthesis, as will be discussed below in this Section. Moreover, although outside the scope of this review, we will also mention the importance of IMCRs in the preparation of large macro- and biomolecular constructs for the ligation and conjugation of biomolecules. Some examples include the ligation of lipids to peptides and oligosaccharides and the ligation of steroids and carbohydrates, as well as fluorescent and affinity tags, to peptides and proteins [78]. This chemistry has also been applied to the site-selective modification of proteins, allowing for the introduction of several payloads onto the antibody trastuzumab, including the cytotoxic drug monomethyl auristatin E (MMAE) [79]. Finally, IMCRs have also been employed for the construction of functionalised materials for drug delivery and other biomedical applications [80].

### 4.1. Passerini Reaction and Its Variants

**Scheme 34:**
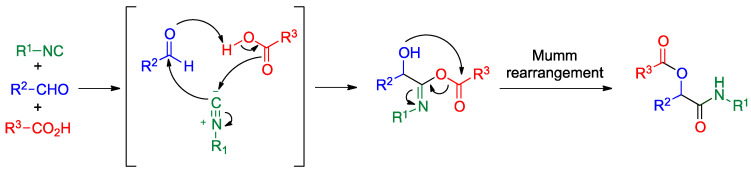
The Passerini reaction and its mechanism.

The first isocyanide-based multicomponent reaction (IMCR) was described in 1921 by Mario Passerini and is based on the combination of an isocyanide, a carboxylic acid and an aldehyde in non-polar solvents to give an α-acyloxyamide. It is normally assumed that the reaction takes place by the concerted mechanism shown in Scheme 34, followed by a Mumm rearrangement [81].

**Scheme 35:**
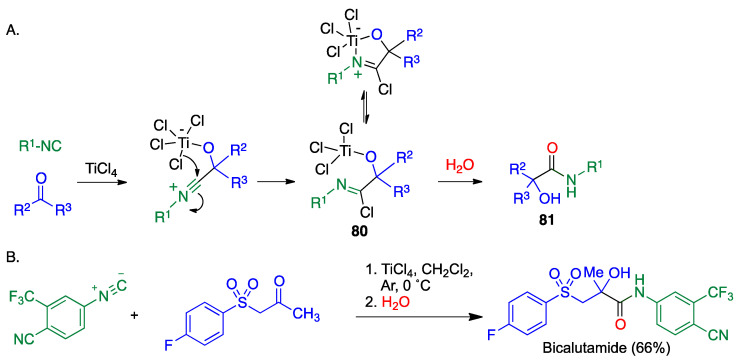
(**A**). The Seebach modification of the Passerini reaction. (**B**). Its application to the synthesis of bicalutamide.

The Seebach variation of the Passerini reaction is carried out in the presence of TiCl_4_, an oxyphilic Lewis acid that coordinates with the ketone and promotes the isocyanide addition [82,83]. The chloroimine intermediate thus formed (**80**) is stabilised by Ti coordination. In this case, the third component is water, which hydrolyses both the chloroimine and the Ti complex and gives an α-hydroxyamide **81** as the final product (Scheme 35A). As an example, we show the application of this method to the synthesis of bicalutamide, an anti-androgenic drug used to treat prostate cancer (Scheme 35B) [51].

**Scheme 36:**
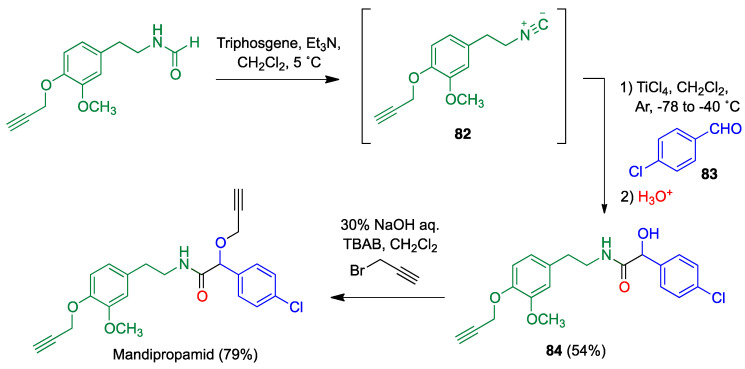
Synthesis of mandipropamid by a Passerini-Seebach approach.

Another example of the use of the Sebaach Ti(IV) variation of the Passerini reaction to obtain marketed bioactive compounds can be found in the synthesis of mandipropamid, an agrochemical compound acting as a fungicide and employed for the control of foliar oomycete pathogens in a range of crops. Intermediate **84** was obtained in 54% yield from the *in situ*-generated isocyanide **83** and *p*-chlorobenzaldehyde. Then, the α-hydroxyamide intermediate **84** was *O*-propargylated to give the target mandipropamid (Scheme 36).

**Scheme 37:**
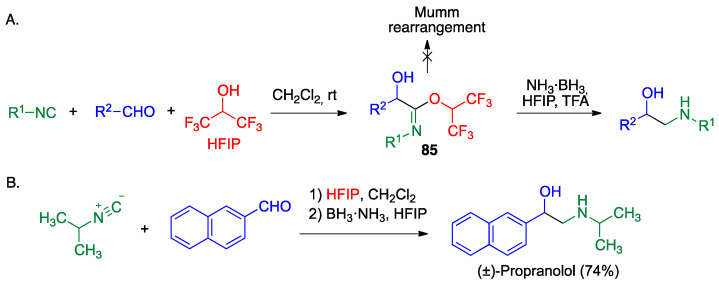
(**A**) The Ruijter modification of the Passerini reaction for the preparation of β-aminoalcohols. (**B**) Its application to the synthesis of racemic propranolol.

In principle, alcohols cannot replace carboxylic acids in the Passerini reaction since they lack an electrophilic site needed for the Mumm rearrangement. However, in 2018, Ruijter reported that hexafluoro-2-propanol (HFIP) could be employed as a surrogate of the carboxylic acid since in this case the intermediate imidates **85**, although unable to undergo the Mumm rearrangement, are sufficiently stable to allow their isolation and can be then reduced to β-aminoalcohols (Scheme 37A) [84]. This was an important discovery due to the widespread presence of this structural motif in drug molecules such as β_2_-adrenergic agonists and β-blockers. This chemistry was employed to obtain racemic propranolol, a β-blocker used to treat hypertension and other cardiovascular diseases, by reaction between naphtaldehyde, isopropyl isocyanide and HFIP as acid surrogate followed by reduction under mild conditions with BH_3_·NH_3_ (Scheme 37B).

**Scheme 38:**
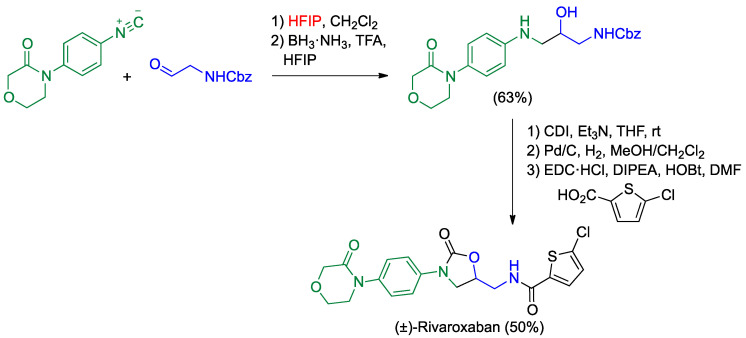
Synthesis of rivaroxaban based on the Ruijter modification of the Passerini reaction.

Another application of this reductive Passerini protocol has allowed a very efficient synthesis of racemic rivaroxaban, a representative member of the new generation of anticoagulants with a mechanism of action based on the inhibition of coagulation factor Xa [84] (Scheme 38).

**Scheme 39:**
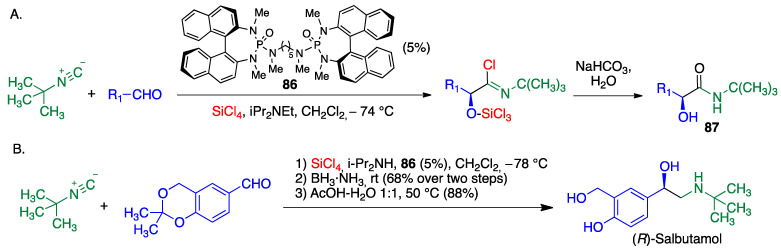
(**A**) Enantioselective truncated Passerini reaction in the presence of silicon tetrachloride. (**B**) Its application to the enantioselective synthesis of (*R*)-salbutamol.

Control of the stereocenter generated in the Passerini reaction is an important issue in API synthesis. The first enantioselective Passerini-type reaction was reported by Denmark [85] and involved the use of silicon tetrachloride instead of the usual carboxylic acid. This compound, acting as a weak Lewis acid, interacts with the chiral bisphosphoramide **86**, forming a silicon cation that activates the aldehyde for nucleophilic attack of the isocyanide from one of the enantiotopic faces of the carbonyl (Scheme 39A). Recently, Virieux and Ayad have applied a combination of this concept and the Ruijter modification of the Passerini reaction to obtain enantiomerically pure β-aminoalcohols **87**, including the (*R*) active enantiomer of the β_2_-adrenergic agonist salbutamol (Scheme 39B) [86].

**Scheme 40:**
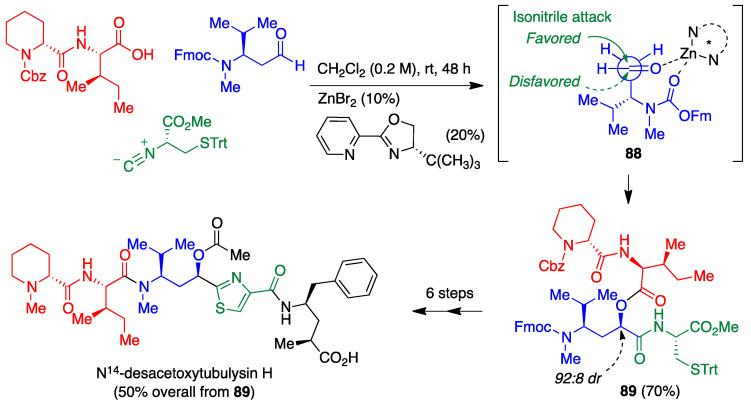
Short synthesis of N^14^-desacetoxytubulysin H based on a diastereoselective Passerini reaction.

Tubulysins are natural tetrapeptides, produced by myxobacteria, that disrupt the microtubule spindle and are among the most potent known antimitotic agents. Some of them have been employed as warheads of clinically tested drug-antibody conjugates, including EC1169 and OMTX705, and a tubulysin-folate conjugate known as EC1456 has also undergone clinical testing as an anticancer treatment. Due to their biological importance, tubulysins are popular synthetic targets, but conventional routes suffer from the need for coupling sterically hindered amino acids and generally require extensive functional group manipulations and chiral separations. Access to these molecules has been greatly simplified by strategic use of a diastereoselective Passerini reaction. Thus, a concise total synthesis of the highly potent N^14^-desacetoxytubulysin H was reported by Dömling [87] and relied on a diastereoselective Passerini reaction in the presence of zinc bromide and a chiral oxazoline additive, which induced a diastereoselective addition of the isonitrile to the aldehyde, via the transition state **88**, to yield compound **89**. This intermediate was transformed into the target molecule in 6 additional steps, starting with a base-promoted Mumm re-arrangement (Scheme 40). Other groups have obtained tubulysin derivatives using a similar approach [88].

### 4.2. Ugi Reaction

After the development of the Passerini reaction, isocyanide-based multicomponent chemistry lay dormant until 1959, when Ivar Ugi expanded the synthetic possibilities of the reaction by introducing a fourth component, namely a primary amine. This process, known as the Ugi four-component reaction (U-4CR) or simply the Ugi reaction, allows the isolation of dipeptide scaffolds, which increased the potential of isonitrile-based multicomponent reactions as tools for drug discovery [89,90].

**Scheme 41:**
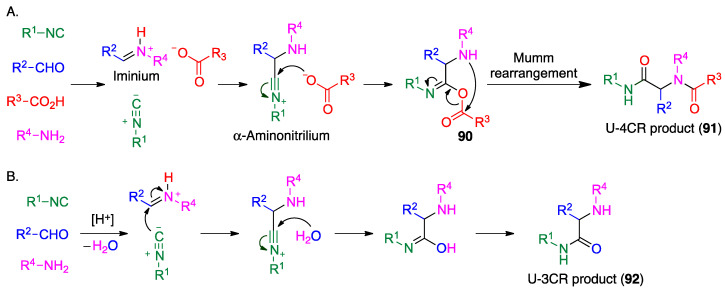
The Ugi-4CR (**A**) and Ugi-3CR (**B**) reactions and their mechanisms.

The Passerini and the Ugi reactions differ in their mechanisms. The Ugi reaction is usually carried out in polar solvents, which suggests the formation of polar ionic intermediates, compatible with a stepwise mechanism. The first step of the classical U-4CR is the condensation between the carbonyl group from an aldehyde or ketone and an amine. Then, the imine thus formed is protonated by the carboxylic acid and the activated iminium reacts with the isocyanide leading to an α-aminonitrilium derivative. The carboxylate attacks this intermediate to give **90** and the amino group promotes the irreversible Mumm rearrangement, which drives the equilibria to the formation of product **91** (Scheme 41A) [91].

A three-component version of the Ugi reaction (U-3CR or truncated Ugi reaction) starts from a carbonyl component (aldehyde or ketone), an amine and an isocyanide to give an α-aminoamide **92**. Although an acid catalyst is needed, it is not incorporated into the final product. The mechanism of the classical U-3CR is initially the same as that of the U-4CR, but, due to the absence of the carboxylic acid component, its role as a nucleophile is played by a molecule of water released in the initial amine-aldehyde condensation (Scheme 41B) [92].

**Scheme 42:**
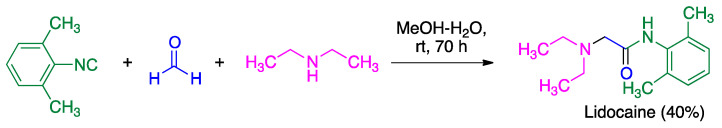
Synthesis of xylocaine by an Ugi-3CR reaction.

The pioneering work by Ugi in isocyanide multicomponent reactions boosted the interest in combinatorial chemistry as a tool in drug discovery [93]. Moreover, due to their high convergence and atom economy, it increased the interest in multicomponent reactions in drug discovery and in synthetic processes of industrial interest [94]. One of the first examples was lidocaine (Xylocaine^®^), a local anaesthetic that acts by blocking the Na^+^ neuronal channels promoting depolarization. This compound was synthetised by an Ugi-3CR procedure in a single synthetic step by combination of dimethylamine, formaldehyde and 2,6-xylyl isocyanide (Scheme 42) [95]. The same chemistry has allowed for the efficient preparation of many members of the amide type of local anesthetics [96].

**Scheme 43:**
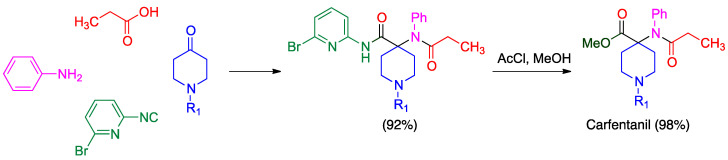
Synthesis of carfentanil by an Ugi-4CR reaction.

Carfentanil, a µ-opioid agonist used as an analgesic and described in Section 2.1, was synthesised by a direct 4CR-Ugi reaction with a 4-piperidone derivative, aniline, propionic acid and 2-bromo-6-isocyanopyridine. After the Ugi reaction, this convertible isocyanide became a cleavable amide that was transformed into the required ester by treatment with acetyl chloride in methanol, affording carfentanil by a transacylation reaction (Scheme 43) [97]. Carfentanil amide derivatives were also synthesised employing the Ugi reaction [98].

**Scheme 44:**
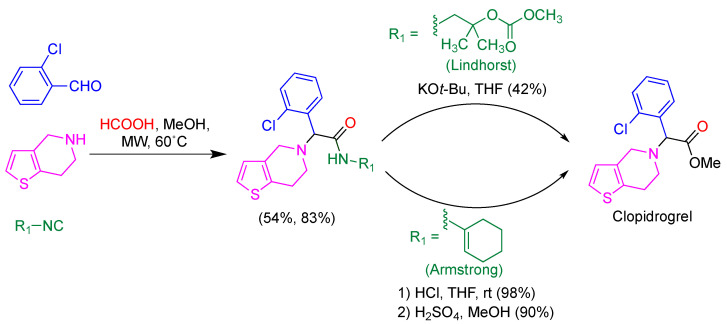
Synthesis of racemic clopidogrel by an Ugi-4CR reaction.

A similar strategy was used to obtain clopidogrel, an inhibitor of platelet aggregation mentioned in Section 2.5. Again, an aldehyde, an amine and a convertible isocyanide were combined in a single synthetic step. The first approach was based on the Lindhorst isocyanide **93** that in basic medium undergoes a rearrangement to form in a single step the suitable ester [51]. An alternative is the Armstrong isocyanide, an enamine-based compound. After the Ugi reaction, the enamide obtained was easily cleaved by treatment with acid and underwent the subsequent transesterification to afford clopidrogrel (Scheme 44) [51].

**Scheme 45:**
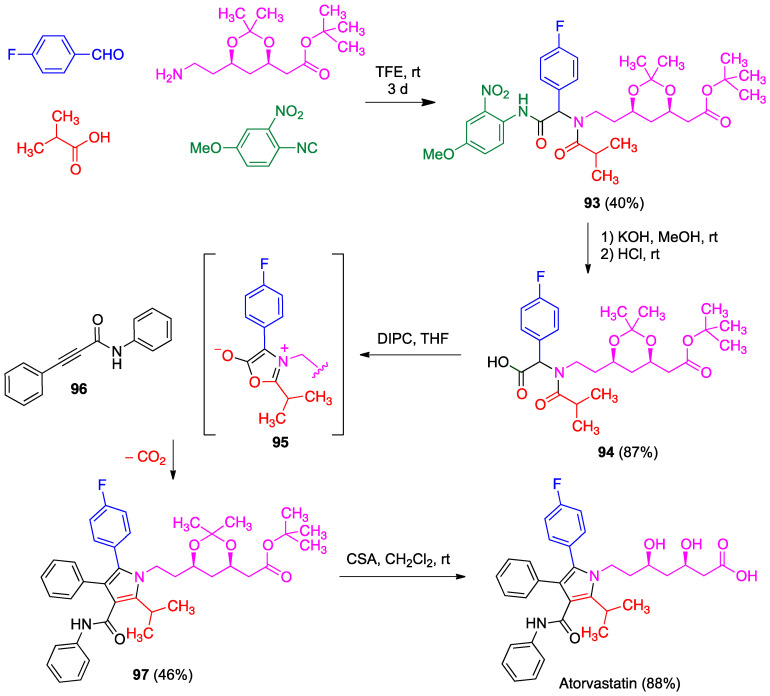
Synthesis of atorvastatin based on an Ugi reaction.

Atorvastatin, previously mentioned in Section 3.2 as a blockbuster drug used to reduce cholesterol levels in plasma, can be obtained by a procedure based on the synthesis of a key intermediate by a 4C-Ugi multicomponent reaction, as shown in 2019 by the Dömling group [99]. Thus, the combination of the suitable amine, aldehyde, carboxylic acid and isocyanide afforded intermediate **93**. Thanks to the electron-withdrawing effect of the nitro group, the amide function could be selectively hydrolyzed to give carboxylic acid **94**. Its diimide activation generated in situ the münchnone derivative **95**, a 1,3-dipole that takes part in a [3 + 2] cycloaddition with alkyne **96** [100] that yields the protected atorvastatin derivative **97**. Finally, camphorsulfonic acid (CSA)-mediated deprotection of the acetal and ester functions afforded atorvastatin (Scheme 45).

**Scheme 46:**
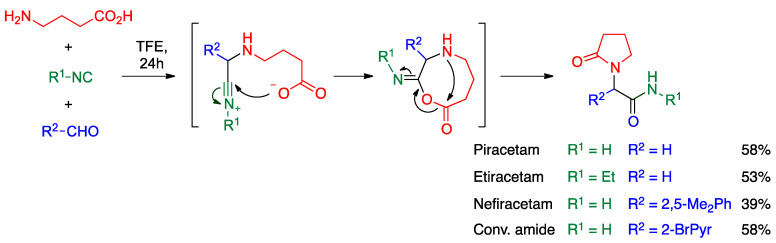
One-step synthesis of several racetam drugs using an Ugi reaction.

Racetams are a family of pyrrolidone-based drugs, relevant in the treatment of several disorders such as anxiety, epilepsy and hypoxia [101], acting as allosteric modulators of glutamate receptors. In 2017, Ruijter, Orru and co-workers [102] described a multicomponent approach to obtain racetam derivatives based on the combination of γ-aminobutyric acid and the suitable isocyanide and aldehyde, with the γ-butyrolactam characteristic of racetams being generated by the Mumm rearrangement. Some examples obtained directly in a single step were piracetam, etiracetam and nefiracetam. Other racetams with diverse amide substitution were obtained through post-modification reactions based on the use of convertible isocyanides such as the 2-bromopyridine derivative (Scheme 46).

**Scheme 47:**
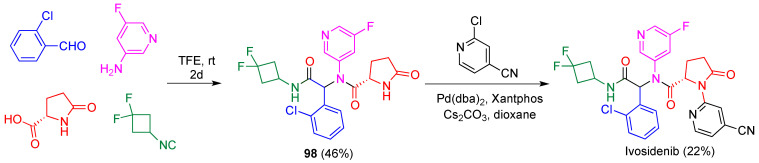
Synthesis of ivosidenib.

The potential of the Ugi reaction to synthetise peptide-like molecules was successfully demonstrated by the synthesis of ivosidenib, an anticancer drug acting by inhibition of isocitrate dehydrogenase-1 (IDH1), an enzyme that is mutated in several forms of cancer. The 4C-Ugi reaction afforded intermediate **98**, which, after a C-N coupling reaction to introduce the cyanopyridine motif in the pyrrolidone NH, gave ivosidenib in moderate yields (Scheme 47) [103].

2,5-Diketopiperazine (DKP) is a cyclic dipeptide found in many natural products, a feature that makes this class of compounds an attractive scaffold in medicinal chemistry. 2,5-Diketopiperazines are readily available from suitably designed Ugi adducts, and we will now discuss some examples of syntheses of drugs containing this simple cyclic peptidic framework, together with others where a diketopiperazine is an advanced synthetic intermediate.

**Scheme 48:**
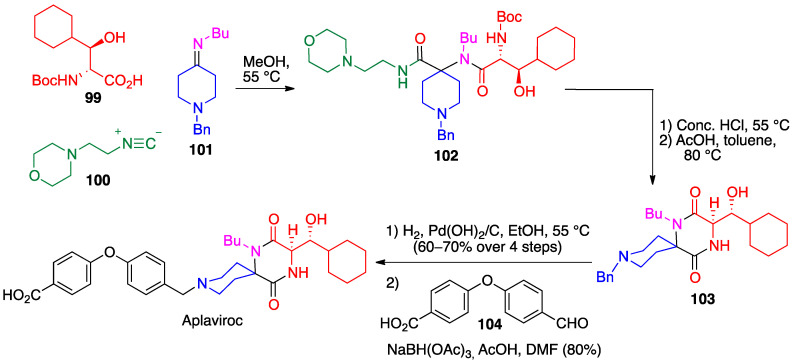
Synthesis of aplaviroc having an Ugi reaction as the key step.

The key step of the synthesis of aplaviroc, a non-competitive CCR5 antagonist with antiviral effect developed for the treatment of HIV, is the reaction between N-Boc-amino acid **99**, isocyanide **100**, and imine **101**, which afforded the Ugi product **102**. Its *N*-deprotection followed by heating in acetic acid induced its cyclization into a 2,5-diketopiperazine **103**, and a final reductive amination step with the phenoxybenzaldehyde derivative **104** yielded aplaviroc (Scheme 48) [104].

**Scheme 49:**
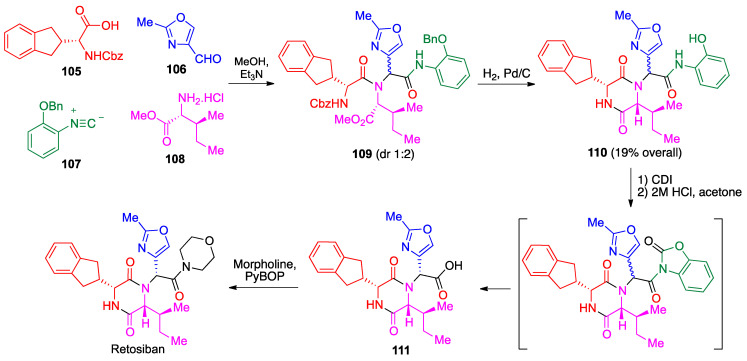
Synthesis of retosiban.

Retosiban (GSK-221,149-A) is an orally active oxytocin receptor antagonist developed by GlaxoSmithKline for the prevention of preterm birth. Its synthesis features an Ugi reaction from the protected (*R*)-indanylglycine derivative **105**, 2-methyloxazole-4-carboxaldehyde **106**, 2-benzyloxyphenylisonitrile **107** and D-allo-isoleucine methyl ester **108**, which yields the linear dipeptidic intermediate **109** as a 1/2 diastereomeric mixture. Catalytic hydrogenation deprotected both the *O*-benzyl and Cbz groups, with concomitant cyclization to generate the DKP ring (compound **110**) [105]. The selective hydrolysis of the phenolic amide was achieved by reaction with carbonyl diimidazole (CDI), followed by acid hydrolysis to give the carboxylic acid **111**, which was transformed into retosiban by activation of the acid with PyBOP (benzotriazole-1-yloxytrypyrrolidinohexafluorophosphate) followed by addition of morpholine. Interestingly, although intermediates **109** and **110** were mixtures of diastereoisomers, during the activation with carbonyl diimidazole/acid hydrolysis sequence the position adjacent to the exocyclic amide epimerised to yield the desired diastereomer [106] (Scheme 49).

**Scheme 50:**
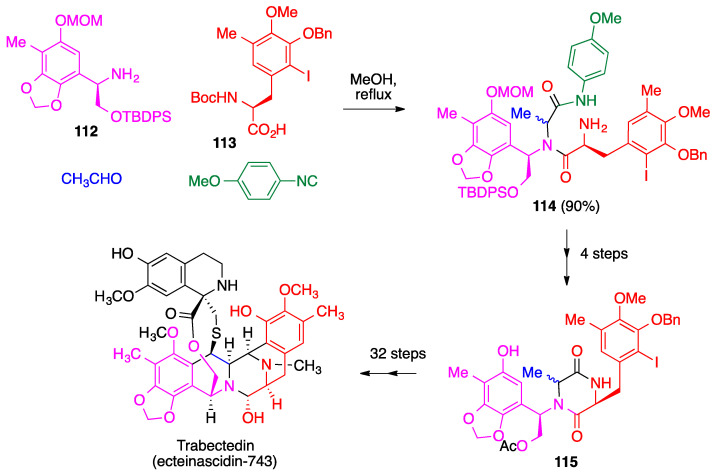
The Ugi reaction featured in Fukuyama’s synthesis of trabectedin.

As a third example of a synthetic route to a highly complex drug molecule having as an intermediate a DKP generated by an Ugi reaction, we will discuss a second synthesis of the anticancer agent trabectedin (see also Section 2.1), developed by Fukuyama [107]. The chiral starting materials **112** and **113**, together with *p*-methoxyphenyl isocyanide and acetaldehyde, furnished dipeptide **114** through an Ugi reaction. This compound contained all of the carbon atoms necessary to form at a later stage the pentacyclic core of the alkaloid and was cyclised in 4 steps to diketopiperazine **115**, which was transformed into the target natural product by a 32-step route that will not be discussed here (Scheme 50).

**Scheme 51:**
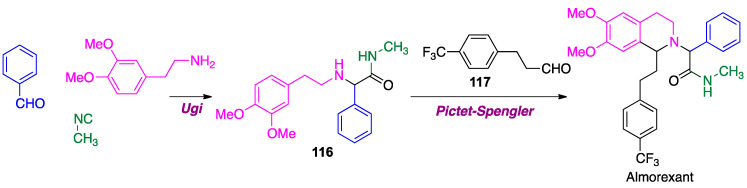
Synthesis of almorexant by an Ugi/Pictet–Spengler sequence.

The Ugi reaction is often combined with post-condensation transformations. One example is the Ugi/Pictet–Spengler sequence that has been employed in some drug syntheses, including that of almorexant, an orexin antagonist developed by GSK to treat insomnia, although its development was discontinued in 2011 due to some liver toxicity issues. An Ugi reaction between 3,4-dimethoxyphenethylamine, benzaldehyde and methyl isocyanide afforded compound **116**, which was the substrate for a Pictet–Spengler cyclisation with aldehyde **117** to furnish almorexant (Scheme 51) [108].

**Scheme 52:**
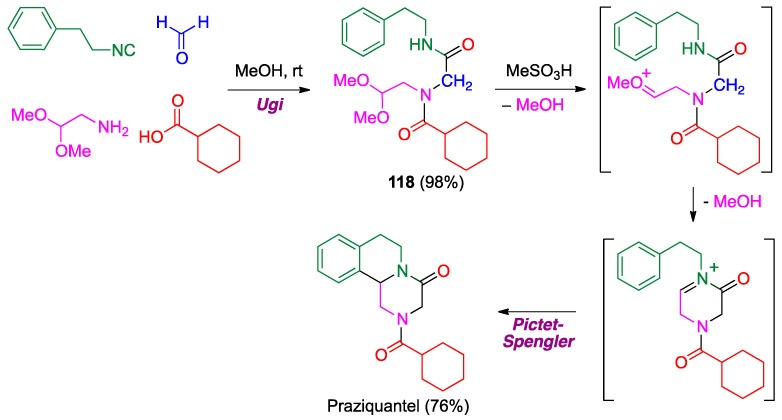
Synthesis of praziquantel by an Ugi/Pictet–Spengler sequence.

A similar approach was used to synthetise praziquantel, an important anti-schistosomal drug, and a library of its analogues [109]. In this particular case, the Ugi reaction involved phenylethyl isonitrile, formaldehyde, 2-aminoacetaldehyde dimethyl acetal, and cyclohexanecarboxylic acid and yielded compound **118**. For the second step, treatment with methanesufonic acid presumably transformed the acetal group into an oxonium intermediate and then into an iminium species that finally underwent the final Pictet–Spengler cyclisation (Scheme 52).

The Ugi reaction normally generates one stereocenter, and there has been much interest in the development of diastereoselective variants of the reaction. The use of chiral auxiliaries for this purpose has been explored in the area of drug synthesis. Thus, (*R*)-lacosamide, a drug used to treat seizures and diabetic neuropathic pain, was obtained based on an Ugi reaction employing a chiral benzylamine derivative as one of the components [110].

**Scheme 53:**
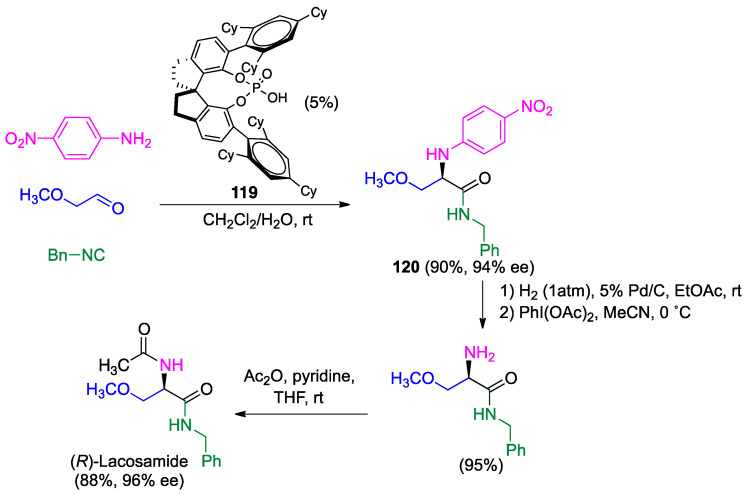
Synthesis of (*R*)-lacosamide by an enantioselective Ugi reaction.

The development of catalytic, enantioselective Ugi reactions has been an unsolved problem for a long time due to the complexity of the reaction system and the existence of competing reactions, but recently organocatalytic methods are showing promise in this regard, and indeed a recent synthesis of (*R*)-lacosamide features an enantioselective Ugi reaction as the key step. Thus, treatment of 4-nitroaniline with 2-methoxyacetaldehyde and benzyl isonitrile in the presence of a chiral SPINOL-based phosphoric acid **119** gave compound **120** with an excellent 94% ee. Nitrophenylamine deprotection and primary amine acetylation completed the synthesis (Scheme 53) [111].

### 4.3. Van Leusen Reaction

**Scheme 54:**
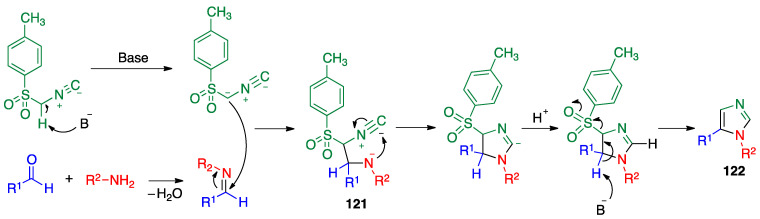
The Van Leusen reaction and its mechanism.

The Van Leusen imidazole synthesis allows for the preparation of imidazole derivatives from aldimines and tosylmethyl isocyanide (TosMIC) under basic conditions [112]. This reaction can also be applied as a sequential multicomponent process, as the aldimine can be previously prepared in situ from an aldehyde and a suitable amine. The aldimine must be formed prior to the addition of the isocyanide since otherwise the aldehyde can react directly with TosMIC to afford an oxazole derivative. The mechanism of the reaction starts with the condensation of the aldehyde with the amine. Then, a base-induced deprotonation of one of the active hydrogens of the isocyanide followed by a Mannich-like addition of the resulting carbanion to the imine gives **121**. This intermediate undergoes a *5-endo-dig* cyclisation followed by the elimination of *p*-toluenesulfonic acid, providing finally a 1,5-disubstituted imidazole derivative **122** (Scheme 54).

**Scheme 55:**
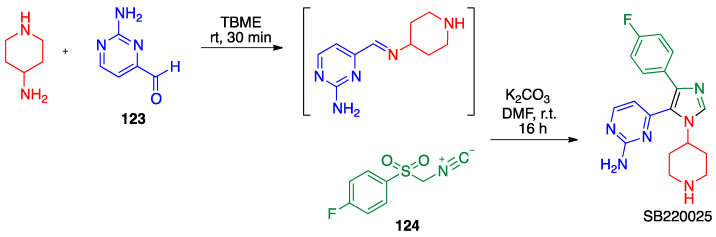
Synthesis of SB220025.

Sisko and Mellinger developed a method to synthesise a library of highly substituted imidazoles through a Van Leusen multicomponent reaction, among which compound SB220025, a potent inhibitor of p38 MAPK, can be highlighted. As this kinase is involved in the inflammatory response, SB220025 was evaluated to assess its potential against inflammatory chronic diseases, reaching phase III clinical trials for the treatment of rheumatoid arthritis. The one-pot protocol for its synthesis consists of a condensation of aldehyde **123** with 4-aminopiperidine in *tert*-butyl methyl ether (TBME), followed by the addition of isocyanide **124** (Scheme 55). A multikilogram method for the synthesis of the required isonitrile **124** was also optimised by the same group [113,114].

## 5. Miscellaneous Named Multicomponent Reactions

### 5.1. Pauson–Khand Reaction

**Scheme 56:**
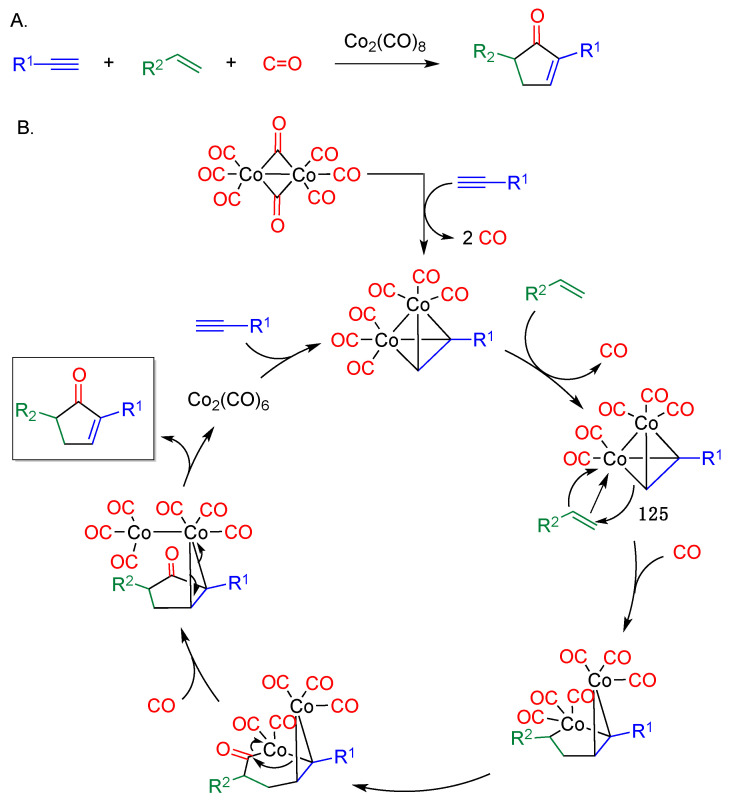
(**A**). The Pauson–Khand reaction. (**B**). Its proposed mechanism.

The Pauson–Khand reaction is a [2+2+1] cycloaddition involving an olefin, an alkyne and carbon monoxide, and constitutes a popular method to access α,β-cyclopentenone derivatives (Scheme 56A). It was unveiled by Peter Ludwig Pauson and Ihsan Ullah Khand in 1970 [115], employing as catalyst a dicobalt octacarbonyl complex, but it remained poorly explored until the 1990s due to difficult purifications and poor yields. Nowadays, many other transition-metal complexes and chiral auxiliaries have been validated to catalyze the reaction and control the stereoselectivity [116].

The mechanistic proposal by Magnus et al., published in 1985, remains the most widely accepted one [117]. As shown in Scheme 56B, the reaction would start with the complexation between cobalt (or any alternative transition-metal), the alkyne and the olefin, forming a metallacyclopentene complex **125**. Carbon monoxide adds to this intermediary complex by insertion into a metal-carbon bond, and a final reductive elimination yields the cyclopentenone product and a dicobalt hexacarbonyl species that readily regenerates the original cobalt complex by reaction with a fresh alkyne molecule.

**Scheme 57:**
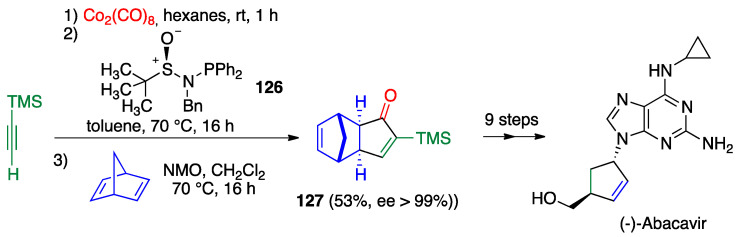
Synthesis of (−)-abacavir featuring an enantioselective Pauson-Khand reaction.

Schmalz et al. [118] reported the synthesis of racemic abacavir, a nucleoside analog-type inhibitor of reverse transcriptase, widely used for the treatment of HIV infection, in which a two-component Pauson–Khand intramolecular reaction was employed. Later, the group of Verdaguer and Riera [119] disclosed an enantioselective synthesis of (−)-abacavir initiated by an enantioselective Pauson–Khand multicomponent reaction between trimethylsilylacetylene, norbornadiene and a dicobalt octacarbonyl complex, assisted by chiral sulfinamide **126** as a chiral auxiliary, obtaining compound **127** in an acceptable yield and excellent enantioselectivity. The reaction was shown to be scalable to multigram quantities with no loss in enantioselection. Nine additional steps, including a retro Diels–Alder reaction, allowed completion of the synthesis (Scheme 57).

### 5.2. Gewald Reaction

**Scheme 58:**
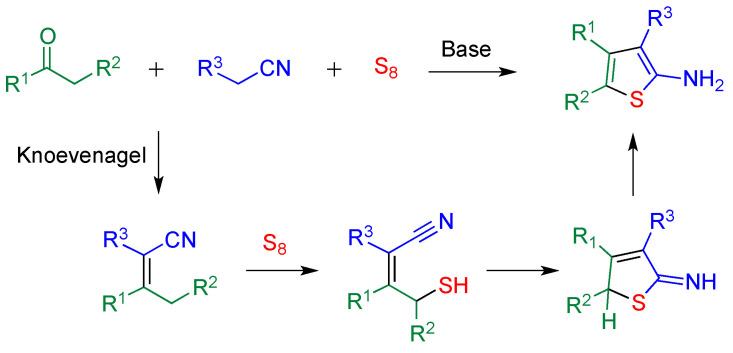
The Gewald reaction and its proposed mechanism.

Karl Gewald, in 1966, reported a three-component reaction from an aldehyde or ketone, a cyanocarbonyl compound and elemental sulfur in the presence of a base to yield 2-aminothiophenes. The scope of this reaction has increased quickly in recent years, and it has become an important methodology in heterocyclic chemistry [120,121]. Its commonly accepted mechanism is shown in Scheme 58 and comprises an initial Knoevenagel condensation, a thionation step, a 5-*exo*-*dig* cyclization, and a final tautomerization.

**Scheme 59:**
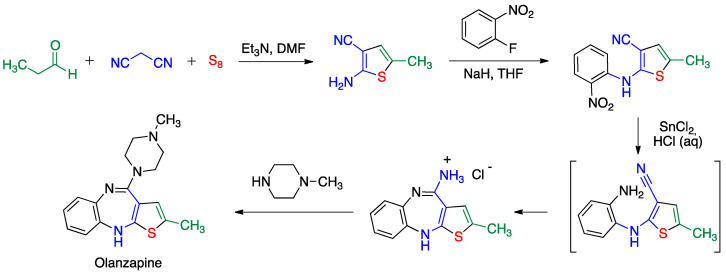
Synthesis of olanzapine from a Gewald product.

Olanzapine, a thienobenzodiazepine derivative, is an important antipsychotic drug useful in the treatment of schizophrenia and psychosis of schizoaffective nature. The first step in olanzapine synthesis is a Gewald reaction from propanal, malononitrile and sulfur in the presence of triethylamine, using DMF as solvent. Three additional steps completed the formation of the tricyclic system [122] (Scheme 59).

**Scheme 60:**
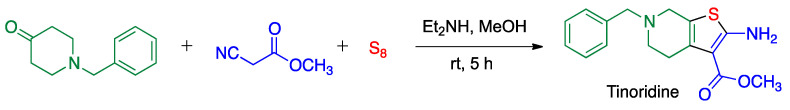
Synthesis of tinoridine.

Another example that illustrates the versatility of the Gewald reaction is the synthesis of tinoridine, a non-steroidal, anti-inflammatory and analgesic agent, from 1-benzylpiperidin-4-one, methyl-2-cyanoacetate and sulfur, using diethylamine as base and methanol as solvent (Scheme 60) [123].

### 5.3. Biginelli Reaction

**Scheme 61:**
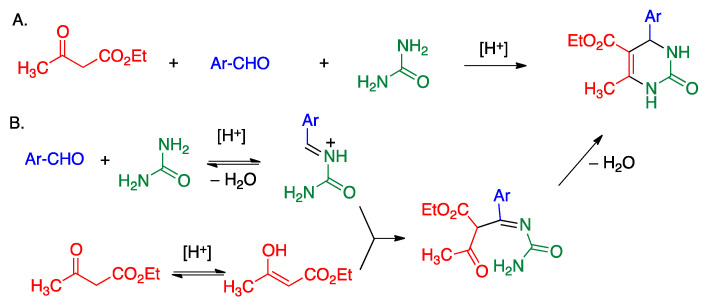
The Biginelli reaction and its mechanism.

The transformation of β-ketoesters, aromatic aldehydes and (thio)urea into dihydropyrimidin-2-(thi)ones, reported by Pietro Biginelli in 1891, is one of the earliest multicomponent reactions and has gained much attention from medicinal chemists due to the fact that it allows the ready preparation of a family of heterocycles of great biological relevance [124]. Its mechanism involves a Knoevenagel condensation between the urea and the aldehyde, followed by Michael addition of the β-ketoester and a final cyclocondensation (Scheme 61).

**Scheme 62:**
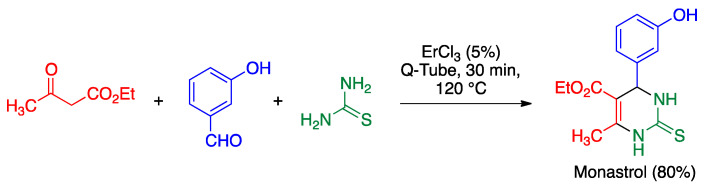
Synthesis of monastrol.

The application of the Biginelli reaction to API synthesis is exemplified in Scheme 61 by the preparation of monastrol, a cell-permeable compound that arrests cells in mitosis by inhibiting Eg5, a member of the kinesin-5 family and has been widely employed to probe the dynamic organization of the mitotic spindle (Scheme 62) [125].

## 6. Miscellaneous Non-Named Multicomponent Reactions

**Scheme 63:**
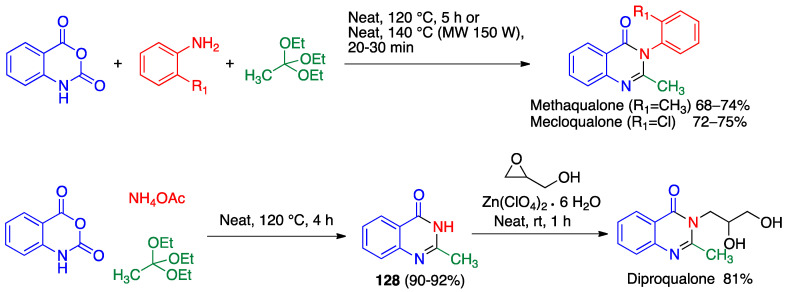
Synthesis of several “qualone” drugs.

The group of Chakraborti disclosed a new multicomponent protocol for the preparation of 2,3-disubstituted quinazolin-4(3*H*)-ones and 2-styryl-3-substituted quinazolin-4(3*H*)-ones and demonstrated its applicability to the synthesis of some drugs belonging to the “qualone” family, such as methaqualone, mecloqualone, and diproqualone [126]. These drugs are agonists of GABA receptors, inducing sedative and anxiolytic effects that have been exploited in medical practice to treat insomnia and anxiety, although their large potential of abuse and use as a recreational drug caused their gradual withdrawal from the market. Thus, the multicomponent reaction of isatoic anhydride, triethyl orthoacetate, and a suitable aniline derivative afforded methaqualone and mecloqualone in good yields. When the multicomponent reaction was performed using ammonium acetate instead of aniline as the amine partner, compound **128** was isolated in good yield and its subsequent treatment with glycidol in the presence of Zn(ClO_4_)_2_·6H_2_O allowed the preparation of diproqualone (Scheme 63).

**Scheme 64:**
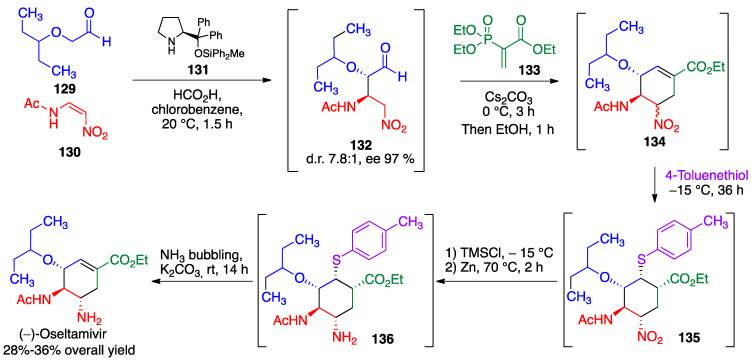
Synthesis of (−)-oseltamivir by a sequential multicomponent process.

Oseltamivir is a neuraminidase inhibitor employed for the prevention and treatment of influenza A and B virus infection. Hayashi et al., interested in the development of multicomponent/one-pot protocols for a green synthesis of complex molecules [127], optimised a one-pot, sequential and enantioselective multicomponent process for the synthesis of (−)-oseltamivir (Scheme 64). The enantioselective Michael addition of α-alkoxyaldehyde **129** to nitroalkene **130**, directed by the prolinol silyl ether derivative **131** as a chiral catalyst, afforded intermediate **132**, which then underwent a domino Michael/intramolecular Horner-Wadsworth-Emmons reaction with phosphonoacetate **133** to yield compound **134**, and then a new Michael addition by 4-toluenethiol to give **135**, with the correct configuration at the α-nitro stereocenter. Nitro reduction and a final elimination step completed the synthesis of enantiopure oseltamivir. This one-pot sequence did not require evaporation or solvent exchange steps and could be performed at a gram scale with a 28–36% overall yield [128].

**Scheme 65:**
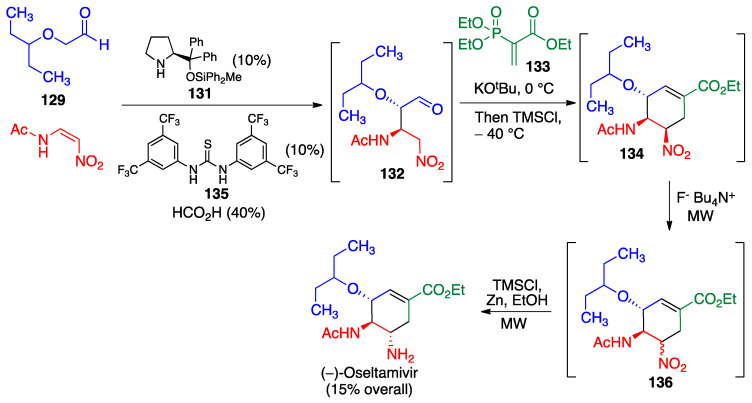
An improved one-pot synthesis of (−)-oseltamivir.

In a more recent refinement of this method that they describe as a “60-min synthesis of oseltamivir”, the Hayashi group [129] has improved the Michael addition step by adding the thiourea catalyst **135** and found a method to achieve the inversion of the stereocenter adjacent to the nitro group without thiol addition, which has resulted in a lower step count. The overall yield of oseltamivir by this protocol is 15% (Scheme 65).

## 7. Combination of Multicomponent Reactions

**Scheme 66:**
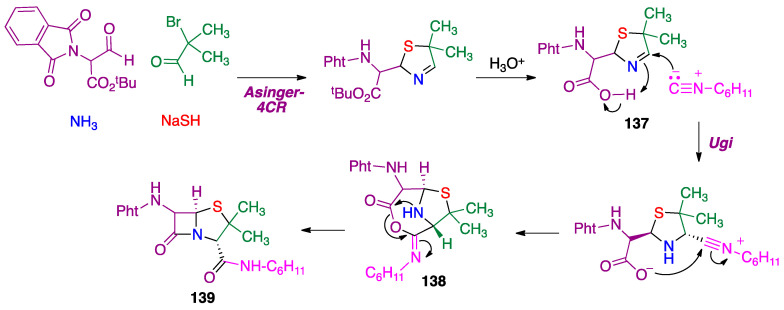
Synthesis of penicillin derivatives based on an Asinger-4CR/Ugi sequence.

The union of two multicomponent reactions is an interesting strategy for the rapid generation of structural complexity [130,131]. One early example of such an approach was reported by Ugi himself [132] and involves an intramolecular Ugi reaction from cyclohexyl isonitrile and the imino and carboxy groups of compound **137**, which in turn had been prepared via an Asinger 4-CR reaction. This transformation afforded the bicyclic system **138**, which readily underwent a Mumm rearrangement to give a penicillin derivative **139** (Scheme 66).

**Scheme 67:**
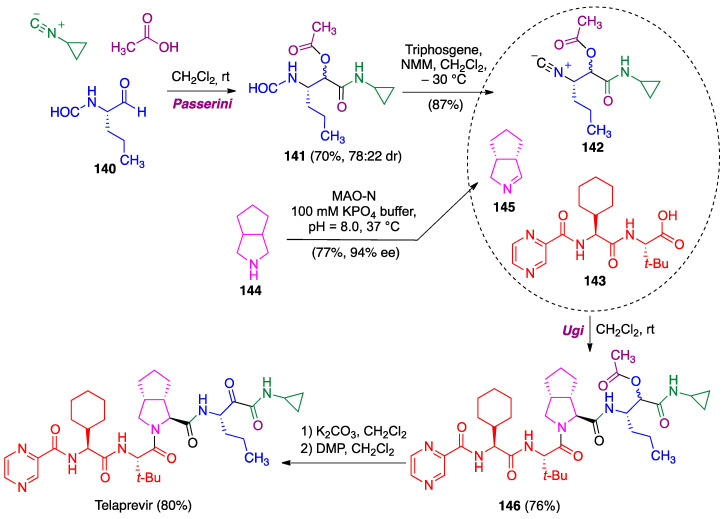
Synthesis of telaprevir combining a Passerini and an Ugi reaction.

An interesting example of the ready assembly of complex peptide-like structures by combining two isonitrile-based multicomponent reactions can be found in a highly convergent synthesis of the anti-hepatitis C drug telaprevir, whose preparation by a different approach was previously mentioned in Section 2.1. This work, reported by Ruijter and Orru [133], combines a Passerini and an Ugi reaction. The initial Passerini reaction was performed from cyclopropyl isocyanide, acetic acid and aldehyde **140**, both generated in situ by Dess-Martin oxidation of the *N*-formyl derivative of commercially available (*S*)-2-amino-1-pentanol. The Passerini product **141** thus obtained was readily dehydrated into the required isocyanide **142** by treatment with triphosgene and *N*-methylmorpholine (NMM), acting as a base. The key Ugi reaction was performed from this isocyanide, carboxylic acid **143** and imine **145**, prepared by chemoenzymatic de-symmetrization of the *meso* bicyclic compound **144** with the enzyme monoamino oxidase N (MAO-N). These three advanced intermediates were reacted together to give the Ugi product **146** in good yield (76%) which, after ester hydrolysis and alcohol oxidation by the Dess-Martin periodinane (DMP), furnished telaprevir (Scheme 67).

**Scheme 68:**
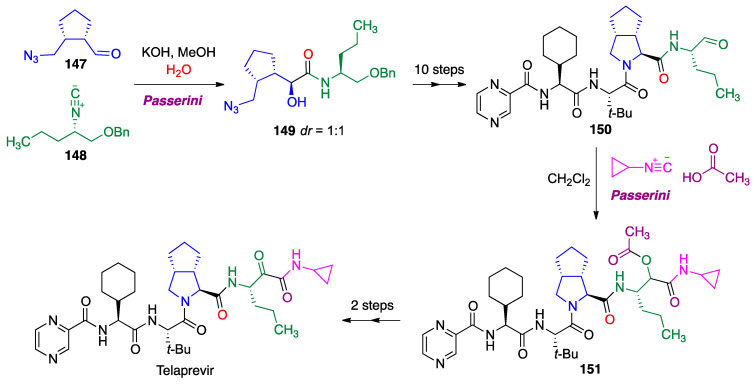
Synthesis of telaprevir involving two Passerini reactions.

An alternative synthesis of telaprevir reported by the Dömling group [134] features two Passerini MCRs. The first one starts from the *in situ*-generated azidealdehyde **147** and isocyanide **148** and afforded compound **149** in 78% yield and in a 1:1 diastereomeric ratio. A ten-step sequence was used to transform this compound into the aldehyde **150**, the substrate for the second Passerini reaction to yield advanced intermediate **151**, whose transformation into telaprevir was achieved in two straightforward steps (Scheme 68).

**Scheme 69:**
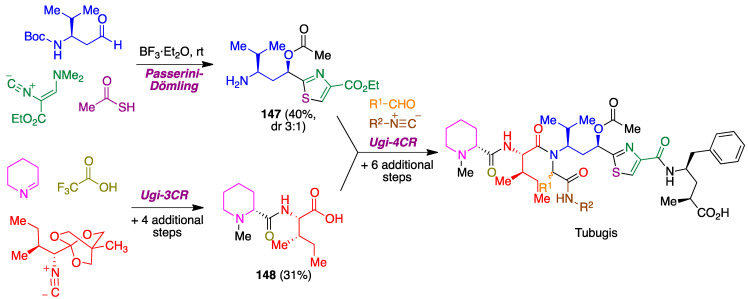
Synthesis of tubugis by combination of three multicomponent reactions.

Tubugis (or tubUgis) are synthetic tubulysin analogues designed to have an N-alkyl amide function that reduces the energy barrier between the *s-cis* and *s-trans* configurations of the amide bond and imparts a greater stability toward enzymatic cleavage. These compounds are readily accesible by a strategy that involves the combination of three multicomponent reactions, as developed by Wessjohann [135]. Thus, a variation of the Passerini reaction described by Dömling afforded the thiazole derivative **147**, while a parallel Ugi 3-CR process afforded **148**. Finally, a second Ugi reaction starting from **147**, **148**, aldehydes and isonitriles afforded the target tubugis derivatives (Scheme 69).

## 8. Summary and Outlook

Multicomponent reactions are widely regarded as one of the most promising approaches to improved sustainability in chemistry. Their advantages over multistep protocols include a higher synthetic efficiency and a reduction in waste generation due to their high atom- and step-economy, which allows for a lower consumption of solvents in extraction and purification stages. The application of MCRs to the preparation of active pharmaceutical ingredients has made good progress in recent years, and we believe that this technology is poised to become one of the mainstays of synthetic chemistry projects in the pharmaceutical industry. Among the pending issues to be resolved in multicomponent synthesis, enantioselectivity is particularly important in view of the relevance of chirality in drug action and therefore in the pharmaceutical industry.

By critically summarizing prior work in this topic, we hope that this review will serve to stimulate further work in this important area of chemistry.

## Data Availability

Data sharing not applicable.
